# Dominating Clasp of the Financial Sector Revealed by Partial Correlation Analysis of the Stock Market

**DOI:** 10.1371/journal.pone.0015032

**Published:** 2010-12-20

**Authors:** Dror Y. Kenett, Michele Tumminello, Asaf Madi, Gitit Gur-Gershgoren, Rosario N. Mantegna, Eshel Ben-Jacob

**Affiliations:** 1 School of Physics and Astronomy, Tel-Aviv University, Tel-Aviv, Israel; 2 Department of Social and Decision Sciences, Carnegie Mellon University, Pittsburgh, Pennsylvania, United States of America; 3 Dipartimento di Fisica e Tecnologie Relative, Università di Palermo, Palermo, Italy; 4 Faculty of Medicine, Tel Aviv University, Tel Aviv, Israel; 5 Israel Securities Authority, Jerusalem, Israel; 6 School of Business and Management, Ben Gurion University, Beer Sheva, Israel; Università del Piemonte Orientale, Italy

## Abstract

What are the dominant stocks which drive the correlations present among stocks traded in a stock market? Can a correlation analysis provide an answer to this question? In the past, correlation based networks have been proposed as a tool to uncover the underlying backbone of the market. Correlation based networks represent the stocks and their relationships, which are then investigated using different network theory methodologies. Here we introduce a new concept to tackle the above question—the partial correlation network. Partial correlation is a measure of how the correlation between two variables, e.g., stock returns, is affected by a third variable. By using it we define a proxy of stock influence, which is then used to construct partial correlation networks. The empirical part of this study is performed on a specific financial system, namely the set of 300 highly capitalized stocks traded at the New York Stock Exchange, in the time period 2001–2003. By constructing the partial correlation network, unlike the case of standard correlation based networks, we find that stocks belonging to the financial sector and, in particular, to the investment services sub-sector, are the most influential stocks affecting the correlation profile of the system. Using a moving window analysis, we find that the strong influence of the financial stocks is conserved across time for the investigated trading period. Our findings shed a new light on the underlying mechanisms and driving forces controlling the correlation profile observed in a financial market.

## Introduction

One clear and immediate conclusion from the financial crisis the world is still trying to recover from, is the need to reshape our knowledge and thinking of the structure and dynamics of financial markets [1]. Over the past few years, a variety of time series analysis methods have been used to study the behavior of stock data for the purpose of detecting some dynamical motifs and stylized facts that describe markets. In the characterization of the correlation profile, these methods usually make use of the basic Pearson correlation coefficient, to investigate stock relationships. Emergent properties, such as the presence of clusters of stocks, have been detected by investigating the correlation between time series of different stock returns [2]–[5].

The presence of a high degree of cross-correlation between the synchronous time evolution of a set of equity returns is a well known empirical fact [6]–[8]. For a time horizon of one trading day, a correlation coefficient as high as 0.7 has been observed for some pair of equity returns belonging to the same economic sector. The Pearson correlation coefficient provides information about how similar is the change in the price of a given pair of stocks. However, the correlation coefficient says nothing about whether a different stock(s) eventually controls the observed relationship between the two stocks. A possible approach to overcome this issue is to make use of the statistical measure of partial correlation [9].

Partial correlation is a powerful tool to investigate how the correlation between two stocks is a result of their correlation to a third mediating stock. For example, suppose we have three stocks, A, B, and C, and we find significant correlation between all three pairs. If we suspect that the correlation between A and B is a result of their individual correlation to C (i.e. A–C and B–C), we suitably remove the (supposed linear) relationship between A and C, and between B and C. We then recalculate the correlation between A and B, which is now the partial correlation, after removing the effect of C. If the resulting partial correlation is significantly smaller than the original correlation, then we can say that the correlation between A and B was mostly due to their individual correlation to C. The use of partial correlations to investigate complex systems is becoming more popular. It has been used in the study of gene networks [10]–[12], and it has also been recently used to investigate how a market index affects the relationships among stocks traded in a market [13]. Partial correlation should not be intended as a causality measure, since many different causal relationships can correlate the same pair of variables. What causal properties can be inferred from studying correlations has been well investigated before [14]–[16]. However, while partial correlation analysis still does not infer causal relationships, it excludes many of the possibilities, and thus is a step in the direction of causal inference.

Causality, and more specifically the nature of the correlation relationships between different stocks, is a critical issue to unveil. The main goal must be understanding the underlying mechanisms of the correlation setting occurring in a stock market. We propose two different methods to accomplish this goal, both methods being based on the construction and analysis of directed networks, based on partial correlations. The first method is a threshold method, where the partial correlation network is constructed by getting rid of all the links associated with a partial correlation smaller than a threshold. The second method selects links among stocks by first ranking the partial correlation according to their intensity and then by choosing a representative set of them satisfying the requirement of a given topological constraint on the resulting network (see below). It is worth noting that partial correlation networks are directed networks, showing the influence of some stocks on the correlation structure of other stocks. Partial correlation networks therefore carry information that is different from that contained in the correlation-based networks, which have been studied in the past [4], [5], [17]–[19].

In this paper, we investigate the daily returns of the 300 largest capitalized stocks traded at the New York Stock Exchange (NYSE) during the time period from January 2001 to December 2003. The capitalization value of stocks was recorded at 12/2003. We choose this system because the emergence properties of this system, as elicited from the analysis of Pearson correlation coefficients, have been thoroughly investigated in the past [3], [4], [13], [17]–[27]. We begin by constructing the partial correlation networks observed for the whole investigated time period. We address these networks as stationary networks. Unlike the case of correlation based networks, these networks reveal the dominant clasp of the financial stocks on correlation structure of the market. We observe that the financial stocks act as the prominent influential force on the correlation structure of other stocks in these networks. Next, in order to investigate the influence of the different stocks for shorter time periods, we perform a dynamical network analysis. In this analysis, we make use of a moving window approach (by using a short time window of one month of trading days, and a larger window of four trading months). This dynamical analysis highlights the fact that the dominant influence of the financial stocks is rather persistent over the studied period.

Our findings provide a unique framework to investigate the underlying backbone of the correlation structure of the market, and reveal the crucial role of the financial stocks in this respect. This observed dominance, and the fact that it is found to be persistent across time, can provide new insights regarding the collapse of financial markets, due to the credit crunch crisis.

## Methods

In this section we illustrate the two partial correlation networks. We start by recalling the definition of partial correlation. A partial correlation coefficient quantifies the correlation between two variables, e.g. stock returns, when conditioned on one or several other variables [9], [13], [20]. Specifically, let 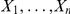 be a sequence of random variables, and 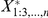 and 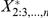 be the best linear approximations to 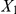 and 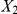 based on 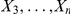. Then the partial correlation coefficient 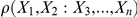 is the correlation coefficient between the random variables 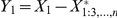 and 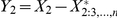, i.e. the correlation coefficient between the residuals of variables 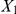 and 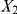. The partial correlation coefficient 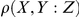 between variables 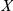 and 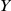 based on the variable 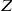 is the Pearson correlation coefficient between the residuals of 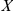 and 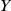 that are uncorrelated with 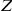. To obtain these residuals of 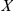 and 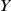, they are both regressed on 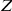.

The number of conditioning variables determines the order of the partial correlation coefficient. For example, 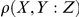 is a first-order partial correlation coefficient, because it is conditioned solely on the 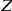 variable [9], [13]. Consider three random variables 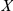, 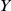, and 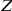. The partial correlation coefficient 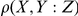 can be expressed in terms of the Pearson correlation coefficients 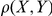, 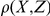, and 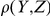 (see for instance ref. [9]) as 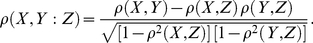
(1)


A small value of 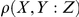 may indicate that variable 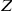 is strongly affecting the correlation between 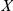 and 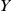, i.e. 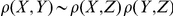. However 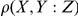 can also be small simply because the Pearson correlation coefficients 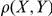, 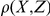, and 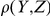 are small, and this is a case that we want to disregard in our analysis. In order to discriminate between these two cases we focus on the quantity 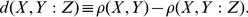
(2)


We address this quantity as correlation influence or influence of 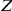 on the pair of elements 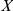 and 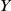. This quantity is large only when a significant fraction of the correlation 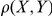 can be explained in terms of 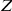. Therefore in the following we shall focus our analysis on large values of 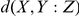.

### Partial correlation networks

There are two main reasons to use partial correlation networks in the description of the influence of specific elements on pair correlations of the system. First of all, partial correlation networks can be seen as filtering procedures that select the most statistically robust information about the influence of specific stocks on the correlation structure of the system. This is analogous to what has been observed in the study of correlation based networks (see for instance [4]). A second reason for constructing partial correlation networks is to simplify the description of the system, which involves 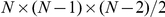 partial correlation interactions according to Eq.s (1,2) when all the available information is considered. In fact partial correlation networks can sometime select a quite small although highly representative number of links.

Let us discuss in detail the two different partial correlation networks we introduce: (i) the Partial Correlation Threshold Network (PCTN), and (ii) the Partial Correlation Planar maximally filtered Graph (PCPG). We consider both these networks, because they lie on rather complementary concepts and their properties can shed light on different aspects of the system. The PCTN is a network where correlation influence values 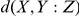 higher than a given threshold, which is specific for each influential stock 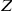, are retained in the network. The PCPG is a network based on hierarchical clustering and it allows one to take into account the heterogeneity of interactions by keeping information in a hierarchical way, so that retaining information about also about poorly interacting groups of elements that could not be selected with a threshold method. It should be noticed that the PCPG method involves a severe filtering of interactions between different elements. In fact it only keeps information about 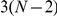 “representative” partial correlations. In the following two subsections we discuss the construction methods and the main properties of both the PCTN and PCPG.

### Partial Correlation Threshold Network

**Figure 1:**
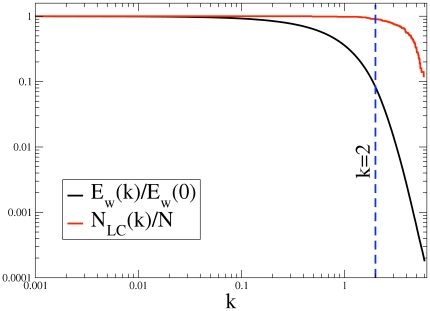
Two measures of PCTN connectivity as function of the parameter 
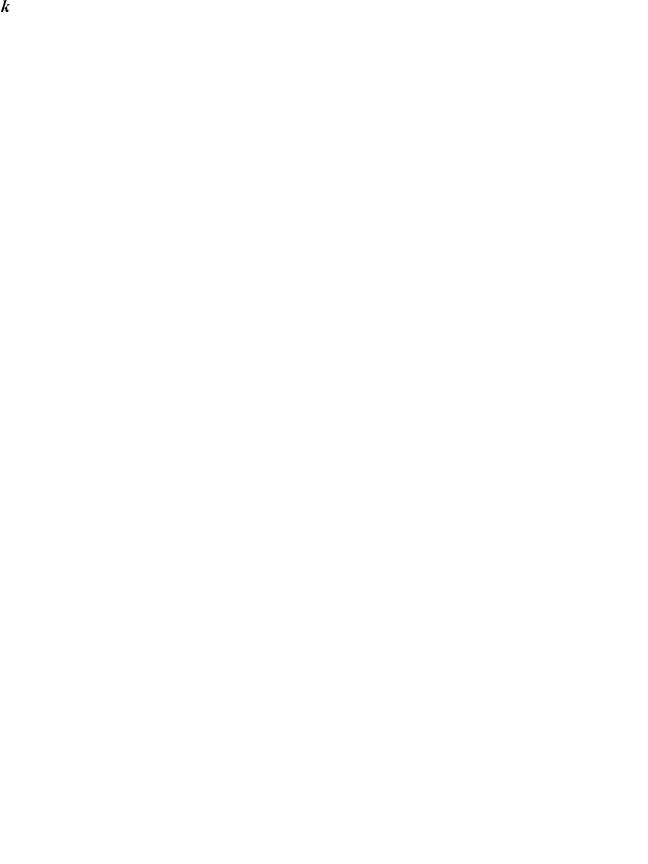
. The value 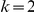 is the one used in the paper.

The PCTN is a network where vertices are the elements of the system, e.g. stocks in our study. Given the elements 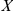, 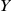, and 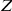, we set two directed links, namely 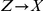 and 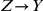, indicating the influence of element 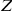 on the correlation between elements 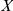 and 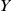, if and only if 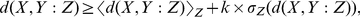
(3)


where 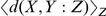 and 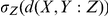 are mean and standard deviation determined with respect to the conditioning element 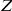, while 
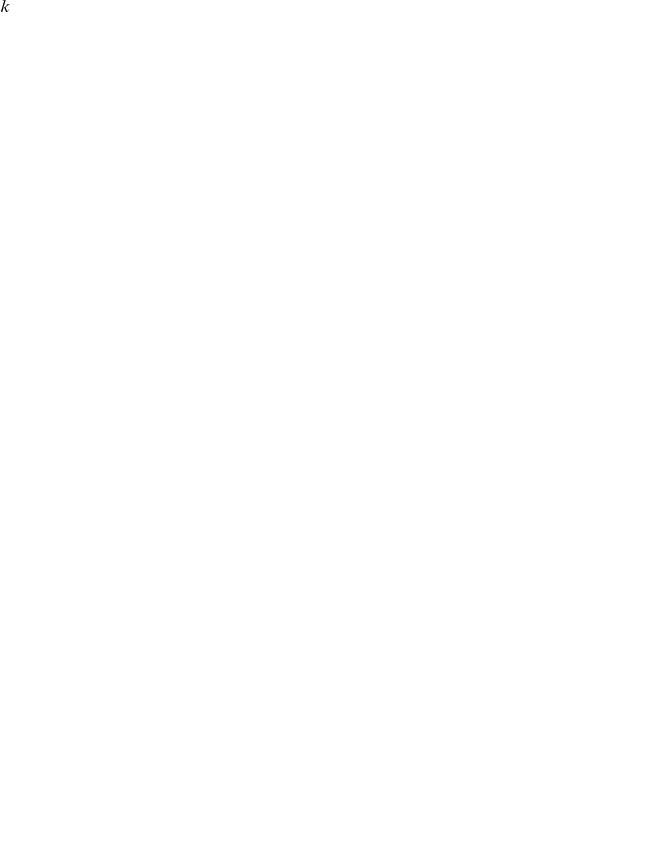
 is a parameter that we name the threshold of influence. The topological and metric properties of the PCTN deeply depend on the value of parameter 
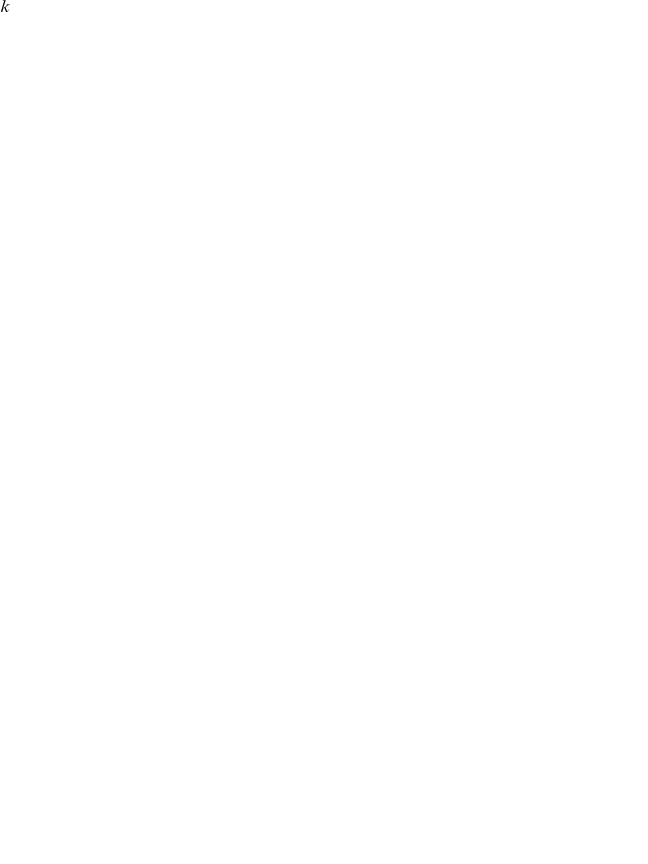
. To the end of selecting a suitable value of 
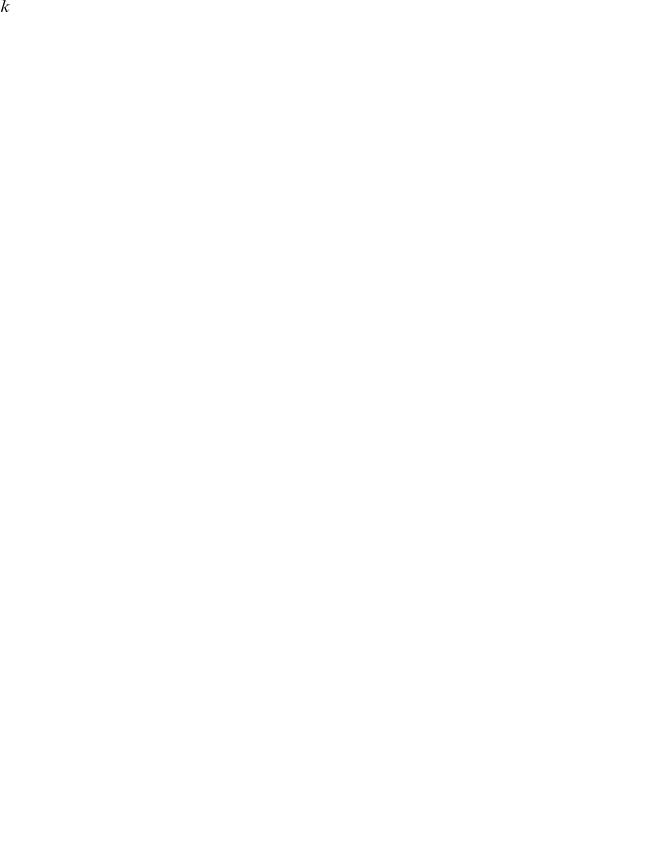
, we iteratively choose different values of this parameter, and compute the sum of the weights of all the edges in the resulting PCTN. We indicate this quantity as 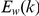. For 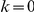 we have 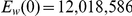. In Fig. 1 we report the fraction 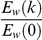 as a function of 
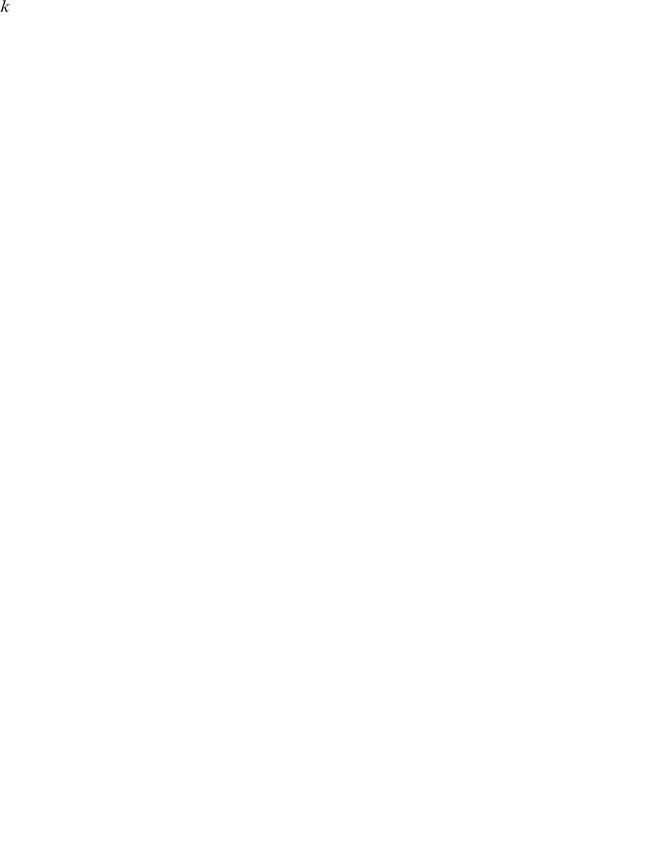
. In addition, we perform a similar analysis for the size of the largest connected component in the network, depending on the value of k. We indicate the total number of vertices in the largest connected component of the PCTN for a given 
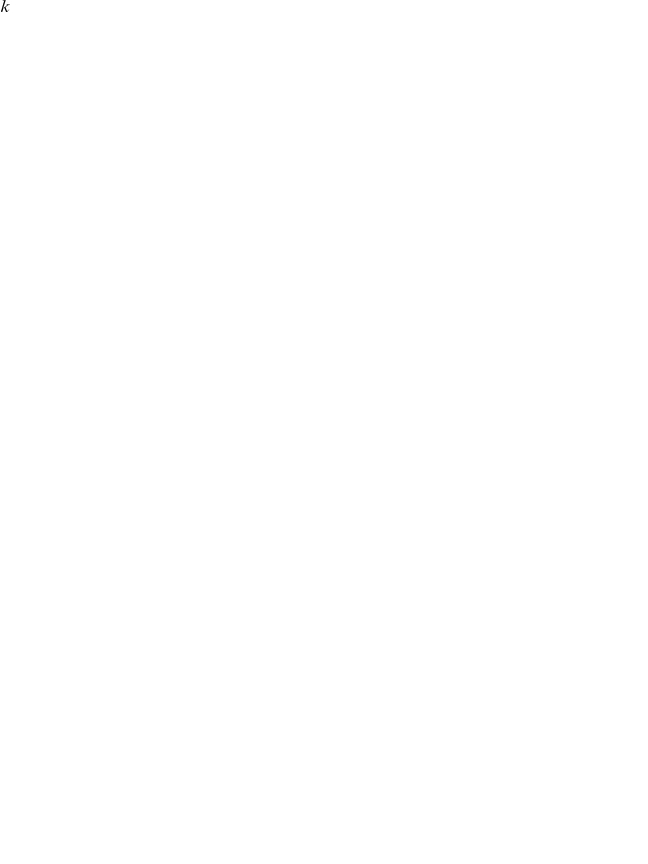
 with 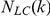. In Fig. 1 we show the quantity 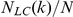, where 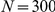 coincides with 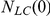. We see from the figure that 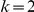 is a good choice, in order to obtain a PCTN with a sizable largest connected component and a non trivial topological and metric properties of the resulting PCTN. The PCTN is a weighted network, in which the weight associated with the directed link 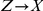 is given by the total number of variables 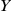 such that Eq. (3) is satisfied. The PCTN is a threshold-based network, and, as well as all threshold-based networks, it is very sensitive to the value of the threshold. At zero threshold, the network is completely connected. As one increases the threshold, the network becomes more informative about the partial correlation structure of the system, but partial correlation selection may be affected by statistical uncertainty. Here we choose a threshold that is sufficiently high so that the PCTN is non-trivial, and sufficiently low so that partial correlation selection does not produce severe filtering. In this way, link selection is not strongly affected by the statistical uncertainty present in partial correlation estimates from finite length time series. Furthermore, while increasing the threshold significantly reduces the number of links in the network, the dominance of the financial sector remains qualitatively the same at higher thresholds.

### Partial Correlation Planar Graph

The PCPG is an adaptation of the Planar Maximally Filtered Graph (PMFG) to deal with asymmetric interactions among the elements of a system. The PMFG is a correlation based network that was introduced in ref. [19]. The PMFG is not a threshold network, and we consider it here because threshold methods might not be able to take into account the heterogeneity of similarities, or influences, that are typically present at different scales of correlation in complex systems. The PMFG is able to tackle such heterogeneity, as well as other correlation based graphs like the Minimum Spanning Tree (MST) [4], [17], [25], which are also based on hierarchical clustering. In fact, both the PMFG and the MST are deeply related to the single linkage cluster analysis. The progressive merging of connected components during the construction of the two networks exactly follows the progressive merging of clusters of the hierarchical tree, which is resulting from single linkage cluster analysis. The MST is included in the PMFG by construction [19]. Both the PMFG and the MST are planar graphs, i.e. they can be drawn on the surface of a sphere without link crossing. The MST is a tree in which the 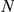 vertices of the network are connected by 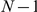 links, while the number of links in the PMFG is 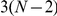. It is to be noticed that 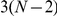 is the maximum number of links allowed to a planar graph, while 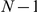 is the minimum number of links allowing a network of 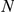 vertices to be connected. In summary, the MST and the PMFG are both planar and connected graphs. The MST has the minimum number of links that must be present in a connected graph, and the PMFG has the maximum number of links allowing to satisfy the planarity constraint. Similarity based networks such as MSTs and PMFGs are informative about the interrelations present among the return dynamics of stocks or assets traded in financial markets [4], [13], [17], [19], [20], [25]–[27]. The advantage of using the PMFG, instead of the MST, is related to its relaxed topological constraint that allows to retain in the graph a larger amount of information carried by the similarity matrix, e.g. loops and cliques of three and four elements, as it is detailed in ref. [19]. The information retained is statistically reliable because links present in the PMFG mostly correspond to the largest pair of similarities/correlations of the system, and this fact guarantees statistical robustness of the network at a very good extent [26].

In order to deal with partial correlations, here we propose an adaptation of the PMFG to the case where interactions among element pairs are not symmetric. We have called this new directed graph the PCPG. It is obtained by starting from the correlation influence 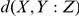. Specifically, we define the average influence 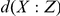 of element 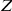 on the correlations between element 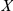 and all the other elements in the system as 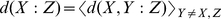
(4)


It is important to notice that in general 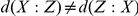. In order to construct the PCPG we list the 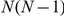 values of the average correlation influence 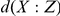 in decreasing order. The construction protocol of the network begins by considering an empty network with 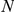 vertices. By starting from the first entry of the list, say 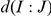, we put a directed link 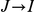 if and only if the resulting network is still planar, i.e. it can be drawn on the surface of a sphere without link crossing [19]. With this choice if 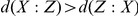 then only the link 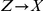 is considered for the inclusion in the PCPG, in order to avoid multiple links and to keep information about the main direction of influence.

The PCPG has a finite number of links, which are 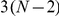 for a system of 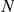 elements. The PCPG turns out to be a quite severe filtering of the 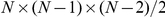 original partial correlation coefficients. In spite of this severe information reduction, it gives a description of the backbone of the system interactions controlling the correlation properties of the system.

## Results and Discussion

We analyze the system of the daily returns time series of the 300 largest capitalized stocks traded at NYSE in the time period 2001–2003. Each stock is classified according to its sector and sub-sector of economic activity. There are 12 different economic sectors of activity, within the classification of stocks of Yahoo Finance (2004) we use. The sectors are: basic material (BM, 24 stocks), consumer cyclical (CC, 22 stocks), consumer non cyclical (CN, 25 stocks), capital goods (CG, 12 stocks), conglomerates (CO, 8 stocks), energy (EN, 17 stocks), financial (FI, 53 stocks), healthcare (HE, 19 stocks), services (SE, 69 stocks), technology (TE, 34), transportation (TR, 5 stocks), and utilities (UT, 12 stocks). The sub-sectors of activity are 80. In Table S1, we provide the list of the 300 stocks, together with the associated sector and sub-sector of activity. The system is investigated in two different ways. The first analysis is performed by considering the whole period of three years under investigation. This analysis gives an overall description of the system, and takes advantage from the length of time series (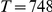 daily records), in order to keep small the statistical uncertainty associated with the partial correlation estimator given in Eq.(1). The second analysis describes the dynamics of influence over time. This is achieved by performing the PCTN and PCPG analysis for shorter time periods in a case with a sliding window approach and in another by considering non overlapping windows. For each time window we compute the partial correlation network and we study the dynamics of influence of individual stocks, as well as economic sectors and sub-sectors of activity, over time.

### Stationary network analysis

The first question we shall answer is about the most influential stocks. As a proxy of influence of a stock 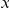 we use the outdegree of the stock in the PCPG, i.e. the total number of directed links outgoing from 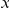 in the network. As a proxy of influence of stock 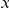 in the PCTN we instead use the weighted outdegree, i.e. the sum of the weights of directed links outgoing from 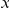 in the network. The rationale behind the choice of using different measures of influence for the two networks lies on the distinct nature of the two networks. In the PCPG, which is a sparse network with only 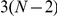 links, information about interactions among the elements of the system is kept in the topology of the network. Therefore using the outdegree to measure the influence of a stock is a good choice, because the outdegree only depends on the topology of the network.

**Figure 2:**
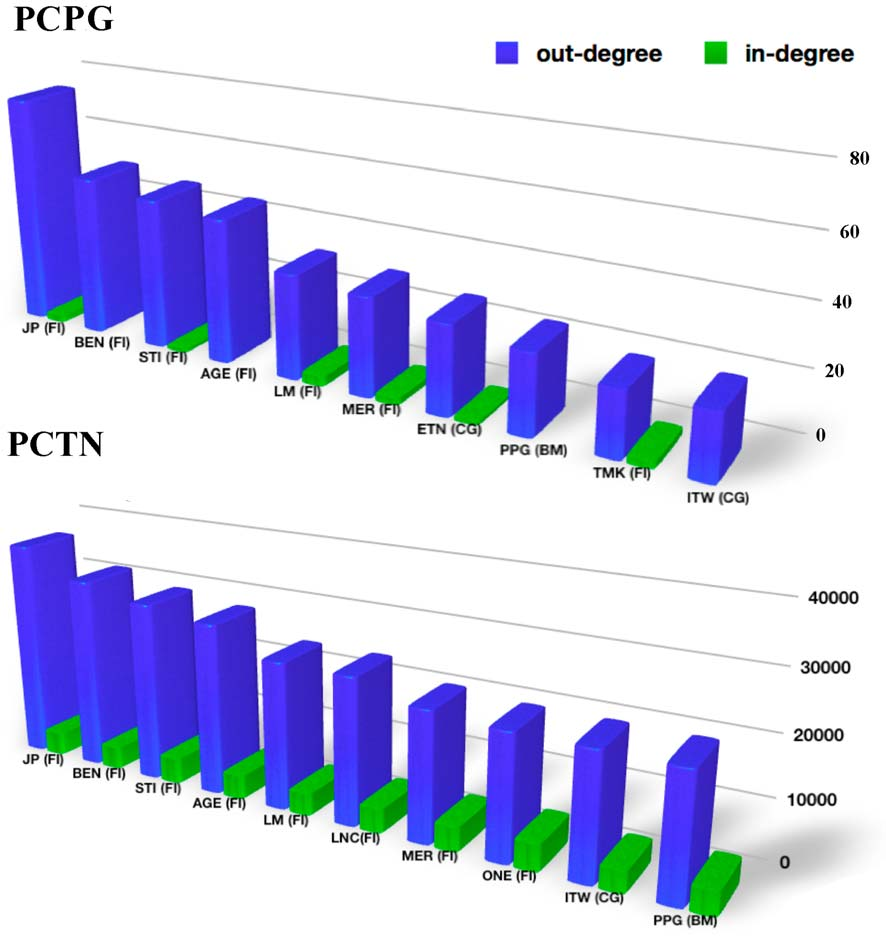
Top ten influential stocks according to the out-degree in both the partial correlation networks.

On the other hand, for low values of the threshold 
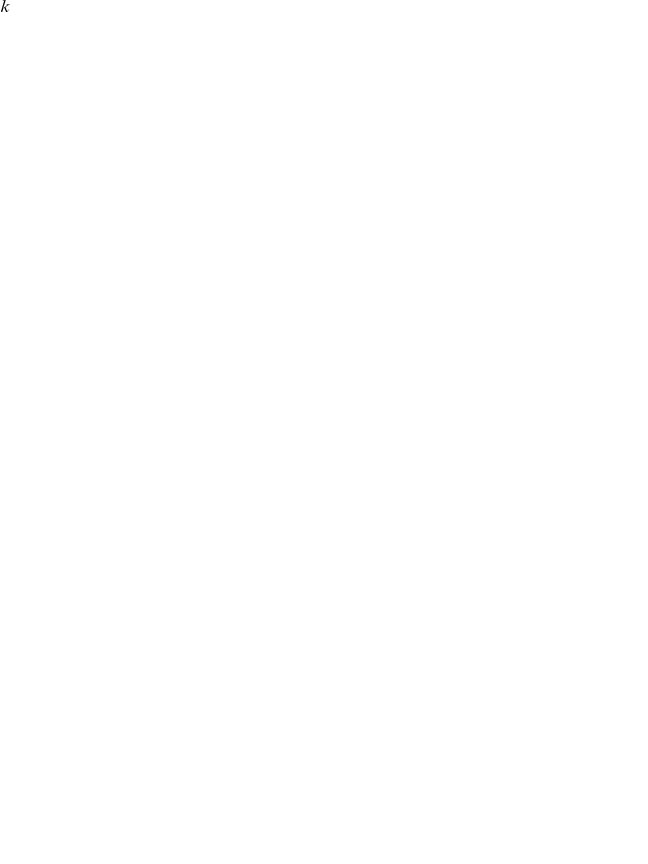
 the PCTN is a quite dense network. For example, when 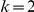 the total number of directed links in the PCTN of the system is 40924, which is of order 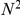. For such a dense network information about relevant interactions in the system is largely kept by link weights and therefore weights need to be taken into account for an appropriate description of network characteristics. In Fig. 2 we report indegree and outdegree of the 10 most influential stocks for both the networks, together with their economic sector of activity. Most of the top 10 influential stocks belong to the financial sector. In order to better understand the mutual influence of economic sectors, in Table 1 we list all the 12 economic sectors of activity, together with some information about their overall influence in the system. The order of economic sectors in Table 1 is according to the outdegree in the PCPG and to the weighted outdegree in the PCTN. The outdegree of a sector 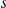 is defined as the total number of links in the PCPG outgoing from stocks belonging to sector 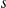 and pointing to stocks belonging to other sectors of activity. We indicate this quantity as 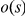. Similarly the quantity 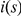 is the indegree of sector s, i.e. the total number of links from stocks not belonging to the sector 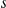 that are directed to stocks belonging to the sector 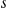. The weighted outdegree 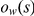 and the weighted indegree 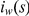 of a sector 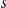, which are used in the PCTN, are defined in a similar way by summing up weights over all links selected as indicated above. Large values of 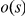 and 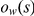 indicate that sector 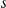 is very influential in the system, while large values of 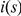 and 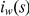 indicate that sector 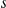 is strongly influenced by other economic sectors of activity. In Table 1 we also report a measure of relative influence of economic sectors based on these indicators. For a given sector 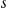, these relative influence measures are defined as: 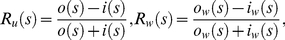
(5)


where the unweighted relative influence 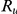 of a sector 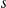 is used in the PCPG, and the weighted relative influence 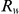 in the PCTN. The relative influence is a quantity ranging in the interval 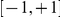. Positive (negative) value of the relative influence of a sector indicates that the sector influences other sectors more (less) than the amount it is influenced by other sectors. Although the financial sector has the highest outdegree in the PCPG and the highest weighted outdegree in the PCTN, its relative influence is quite different in the two cases. Table 1 shows that for most sectors the sign of relative influence 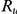 or 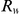 is the same in both networks although the observed value can be quite different. Furthermore, the ranking of sectors according to the outdegree is different for the two networks, with only the financial sector (top) and transportation sector (bottom) ranked the same for both networks. The differences between the rankings of outdegree and between the relative influence of sectors in the networks are probably due to the different information contained in the two networks. The PCPG focuses on the influence of the stock averaged over the entire market whereas a similar constraint is not present in the PCTN. In three cases the sign of the relative influence is opposite in the two networks. This three sectors are energy, utilities and transportation suggesting that the influence of stocks of these sectors might be quite localized. However, in spite of these differences, the correlation between the relative influence values in the two networks is 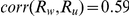. This number is quite high, indicating a similar overall description of the relative influence of different sectors in the two networks.

**Table 1:** Influence of economic sectors according to the outdegree in the partial correlation networks.

	PCTN				PCPG			
rank	sec.	w-outdegree	w-indegree	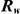	sec.	outdegree	indegree	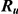
1	FI	8344	4837	0.27	FI	304	4	0.97
2	SE	5248	7663	−0.18	CG	56	17	0.53
3	BM	3727	2743	0.15	CO	38	22	0.27
4	EN	3219	1836	0.27	BM	26	25	0.02
8	CG	2236	1652	0.15	SE	16	136	−0.79
7	CC	2230	2775	−0.11	TE	12	87	−0.76
6	UT	2090	1447	0.18	CC	8	52	−0.73
5	CN	2004	3108	−0.21	EN	6	9	−0.20
9	TE	1904	3797	−0.33	CN	6	53	−0.80
10	CO	1424	1142	0.11	HE	3	44	−0.87
11	HE	988	2625	−0.45	UT	1	18	−0.89
12	TR	882	671	0.14	TR	0	9	−1.00

**Figure 3:**
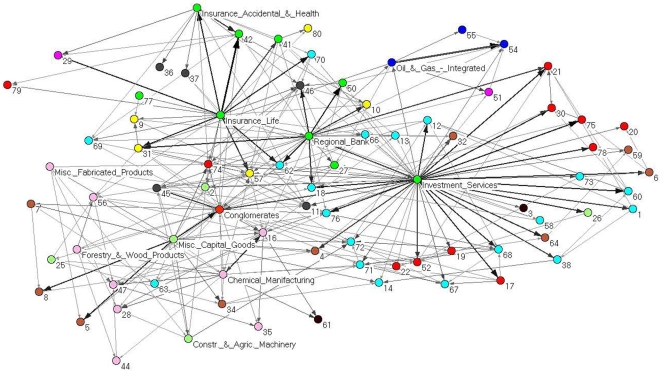
PCPG analysis of the 300 stocks, grouped by their corresponding sub-sector. In this network we present how each sub-sector is affecting the other sub-sectors. The color of vertices is according to the economic sector each sub-sector belongs to. Specifically, basic materials (violet), capital goods (light green), conglomerates (orange), consumer cyclical (tan), consumer non cyclical (yellow), energy (blue), financial (green), healthcare (gray), services (cyan), technology (red), transportation (brown), and utilities (magenta). Sub-sectors with a positive relative influence 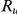 according to Eq.(5) are labeled in the figure. Sub-sectors labeled with numbers are listed in Table S2. We find two main hubs in the network - the investment services and the insurance life sub-sectors. The thickness and gray level of links is proportional to the logarithm of the weight of the link.

By looking at both Fig. 2 and Table 1, it is evident that the financial sector plays a key role in the system. However such relevance could be due to some specific economic sub-sector of activity. In other words, there could be heterogeneity of behavior also inside the sector. In order to better understand the role of economic sub-sectors we analyze the partial correlation networks by merging together all the stocks belonging to the same economic sub-sector of activity in a single vertex of a new sub-sector PCPG. The result is a weighted directed network in which each vertex correspond to a specific economic sub-sector of activity, and the weight of a directed link from sub-sector 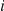 to sub-sector 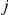 is given by the total amount of directed links outgoing from stocks belonging to sub-sector 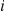 and incoming into stocks of the sub-sector 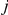 in the PCPG of stocks. In Fig. 3 we show the PCPG of economic sub-sectors. We note from the figure that there are three central sub-sectors of the financial sector in the network. They are (i) Investment services, (ii) Insurance Life and (iii) Regional Banks sub-sectors. These three sub-sectors influence many of the other sub-sectors in the network, and play a major role in the topology of the sub-sector network. It is to notice that such a prominent role of the financial sector and of some of its sub-sectors does not emerge in standard correlation analysis of stock returns at NYSE. A major difference between the economic information carried by standard correlations and the one carried by partial correlations is observed by comparing the role of economic sectors in the corresponding planar networks. The PMFG associated with standard correlations is an undirected network with 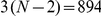 links, i.e. with the same number of links observed in the directed PCPG. The total number of links bridging stocks belonging to different economic sectors is 283 in the PMFG, while this number reaches 476 in the PCPG. This fact indicates that the mutual influence of stocks according to partial correlations is not localized within economic sectors, as it is mostly for standard correlations, but it is spread over the whole partial correlation network. An even more striking difference between standard correlation and partial correlation can be observed by looking at the specific relevance of each economic sector in the planar networks. In Table 1 we report the indegree and outdegree of each economic sector in the PCPG. We note that the outdegree of the financial sector is 304, while its indegree is 4 in the PCPG. On the other hand, the degree of the financial sector is just 119 in the standard correlation PMFG. This finding shows that the influence of the financial sector in PCPG is about 3 times larger than its influence in the standard correlation PMFG. A rather opposite behavior is observed for the services sector of activity. The degree of the services sector is just second to the financial sector in both the planar networks. Its degree is 85 in the standard correlation PMFG, whereas it is 152 in the PCPG. The degree 152 of the services sector can be disaggregated in terms of indegree and outdegree in the PCPG (see Table 1). Its indegree is equal to 136, while its outdegree is just 16. This result shows that the services sector is strongly influenced by other sectors, while it is poorly influential for the whole system. This behavior is exactly the opposite than what has been observed for the financial sector, and this crucial difference between Financial and Services sectors cannot be inferred by looking at networks obtained by using standard correlation as a similarity measure. For the sake of comparison, in the next subsection we show the sub-sector network associated with the standard correlation PMFG, and we list the sector degree in the PMFG.

### Comparison between the PCPG and the PMFG

**Figure 4:**
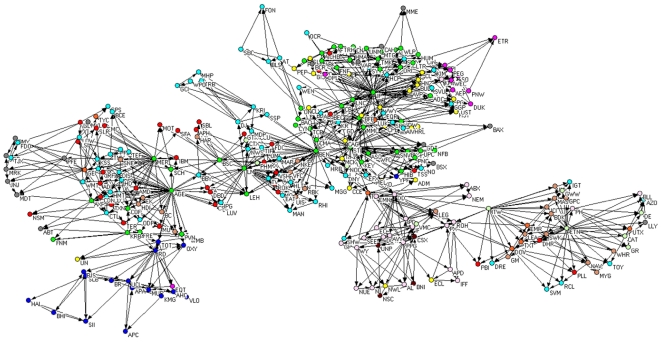
PCPG (partial correlations) of the 300 stocks. Colors of vertices in the network are chosen according to the economic sector each stock belongs to. Specifically: basic materials (violet), capital goods (light green), conglomerates (orange), consumer cyclical (tan), consumer non cyclical (yellow), energy (blue), financial (green), healthcare (gray), services (cyan), technology (red), transportation (brown), and utilities (magenta).

**Figure 5:**
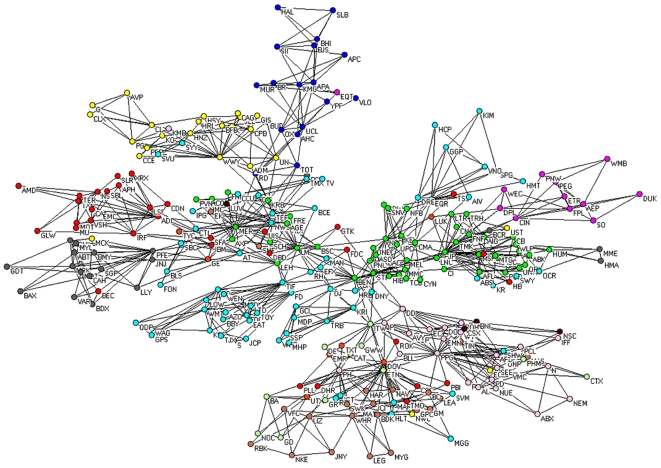
PMFG (standard correlations) of the 300 stocks. Colors of vertices in the network are chosen according to the economic sector each stock belongs to. Specifically: basic materials (violet), capital goods (light green), conglomerates (orange), consumer cyclical (tan), consumer non cyclical (yellow), energy (blue), financial (green), healthcare (gray), services (cyan), technology (red), transportation (brown), and utilities (magenta).

In Fig. 4 we present the PCPG of the 300 stocks. The list of the 300 stocks, together with the corresponding sector and sub-sector of activity, is reported in separate pdf file in the SI. Each node in this network is a single stock, and links are directed from the influential stock to the influenced stock. At this level of hierarchy, distinct hubs appear, and a close inspection of these hubs shows that they are stocks belonging to the Financial sector. Colors of vertices in the network are chosen according to the economic sector each stock belongs to. For the sake of comparing standard correlations with partial correlations, we also report the PMFG constructed from standard correlations of the 300 stock returns in Fig. 5. We remind that the total number of links in both the PMFG and the PCPG is 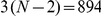.

#### Networks of economic sub-sectors

**Figure 6:**
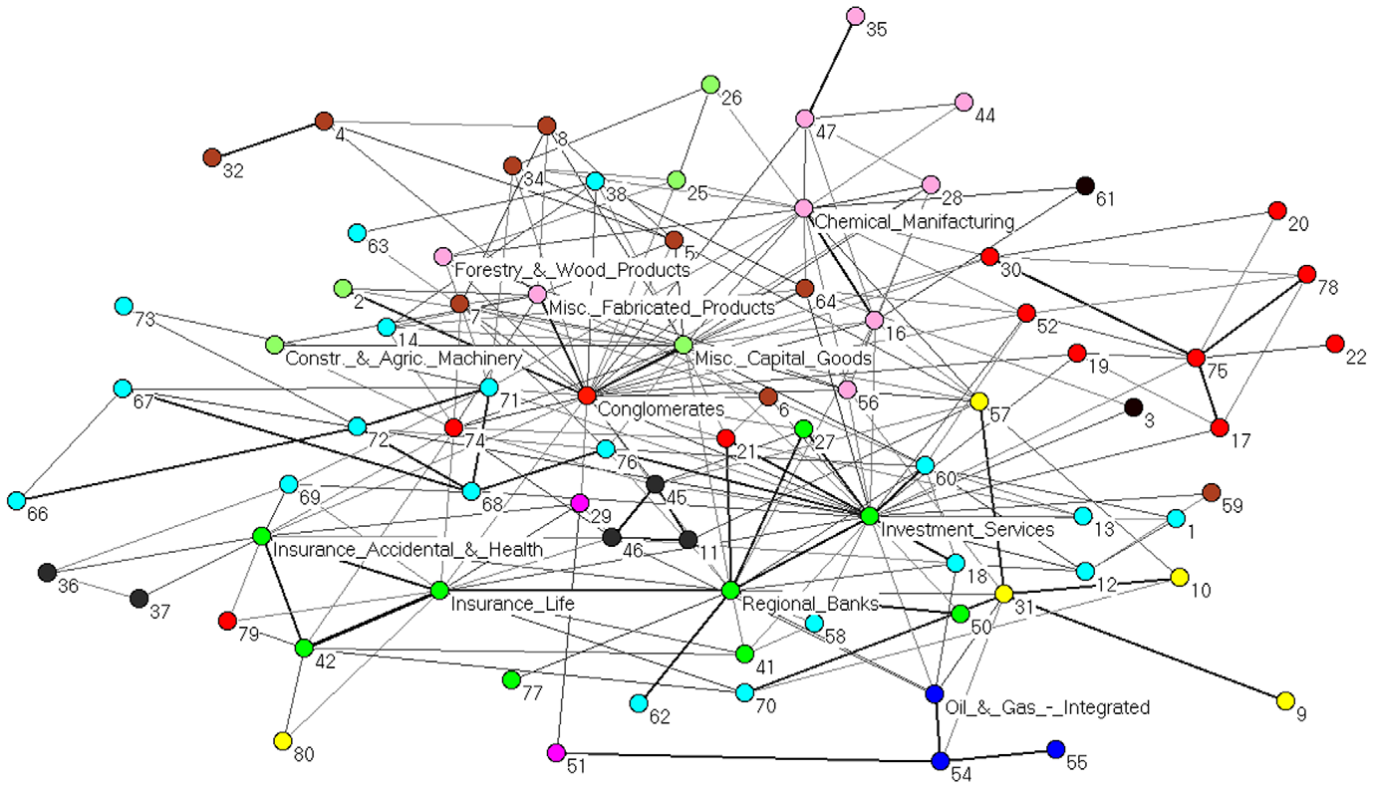
Sub-sector (undirected) network associated with the PMFG (standard correlations). The thickness and gray level of links is proportional to the logarithm of the weight of the link. The color of vertices is according to the economic sector of activity.

We compare some properties of the PMFG, which is based on standard correlations, with the properties of the PCPG associated with partial correlations among the 300 stocks in the system in terms of the relations among sub-sectors of activity. The list of sub-sectors, together with the corresponding sector of activity, is available in Table S2. In Table 2 we report the 5 economic-sub-sectors with the highest weighted degree in the PMFG and with the highest weighted outdegree in the PCPG. It is worth noting that the outdegree of the Invesment services sub-sector is 174 in the PCPG, which is more than twice its degree in the PMFG (77). In Fig. 6 we show the sub-sector network obtained from the PMFG. This figure can be directly compared with the sub-sector network obtained from the PCPG, as reported in Fig. 3. The color of vertices in the figures corresponds to the economic sectors: basic materials (violet), capital goods (light green), conglomerates (orange), consumer cyclical (tan), consumer non cyclical (yellow), energy (blue), financial (green), healthcare (gray), services (cyan), technology (red), transportation (brown), and utilities (magenta). In Table 3 we list the weighted degree of economic sectors in the PMFG and in the PCPG. Please notice that the outdegree of the financial sector in the PCPG is almost 3 times larger than its degree in the PMFG.

**Table 2:** Top 5 sub-sectors according to weighted degree in the PMFG and in the PCPG.

	standard correlation: PMFG			partial correlation: PCPG			
rank	sub-sector	sec.	w-deg.	sub-sector	sec.	w-outdeg.	w-indeg.
1	Investment Services	FI	77	Investment Services	FI	174	3
2	Regional Banks	FI	59	Insurance Life	FI	87	3
3	Conglomerates	CO	55	Regional Banks	FI	76	12
4	Insurance Life	FI	43	Misc. Capital Goods	CG	48	1
5	Misc. Capital Goods	CG	41	Conglomerates	CO	38	22

**Table 3:** Economic sectors: weighted degree in the PMFG and PCPG.

	standard correlation: PMFG		partial correlation: PCPG		
rank	sector	w-degree	sector	w-outdegree	w-indegree
1	FI	119	FI	304	4
2	SE	85	CG	56	17
3	BM	60	CO	38	22
4	CO	55	BM	26	25
5	CG	53	SE	16	136
6	TE	51	TE	12	87
7	CC	49	CC	8	52
8	CN	29	EN	6	9
9	HE	24	CN	6	53
10	EN	15	HE	3	44
11	UT	11	UT	1	18
12	TR	9	TR	0	9

#### Networks of economic sectors

**Figure 7:**
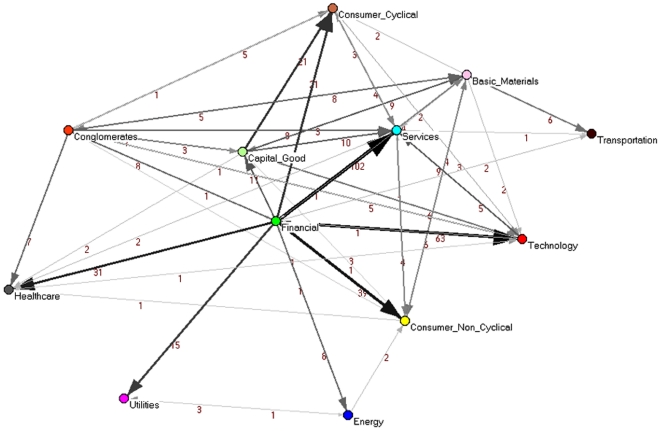
Sector (directed) network associated with the PCPG (partial correlations). The thickness and gray level of links is proportional to the logarithm of the weight of the link. Links are labeled according to the weight. The color of vertices is according to the economic sector of activity.

**Figure 8:**
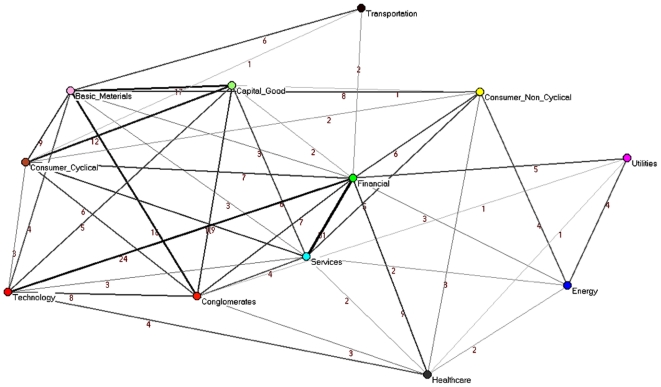
Sector (undirected) network associated with the PMFG (standard correlations). The thickness and gray level of links is proportional to the logarithm of the weight of the link. Links are labeled according to the weight. The color of vertices is according to the economic sector of activity.

We study higher scales of hierarchy in the network, by repeating the comparison of the PCPG and PMFG by grouping stocks at the level of economic sector of activity. This results in networks with 12 nodes, where each node represents a sector. In this network, we calculate how each sector influences the other sectors. The directed network of sectors for the PCPG is reported in Fig. 7, while the undirected network of sectors for the PMFG is shown in Fig. 8. We label links in both networks according to their weight, i.e. according to the total number of stocks of one sector that are linked to stocks belonging to the other sector.

Standard correlations account for the mutual linear influence of stock returns. Partial correlations, instead, account for the influence of a stock into the correlation between the returns of other two stocks. Following this reasoning, one could be tempted to explain the prominent influence of the financial sector in the partial correlation networks as a consequence of the fact that stocks belonging to this sector could preferentially mediate the influence of a financial index in the system. A similar hypothesis can be formulated by saying that partial correlations among stocks can be fairly explained in terms of a single index model. In the next subsection we show that a single index model does not explain all our findings and gives only a rather poor description of sample partial correlations empirically observed. In the next subsection, we also show that a more sophisticated model based on the part of the correlation matrix information selected according to the Random Matrix Theory (RMT) [28] is much more suitable than the single index model to describe empirical partial correlations.

### Factor models

We compare the performance of two distinct factor models in reconstructing the sample partial correlations, which are empirically observed among the 300 stock returns. The first model we consider is the single index model, which is a widespread model in finance. The second model is a model using information associated with a large number of eigenvalues of the sample correlation matrix, the selected eigenvalues being chosen by using RMT.

#### Single index model

**Figure 9:**
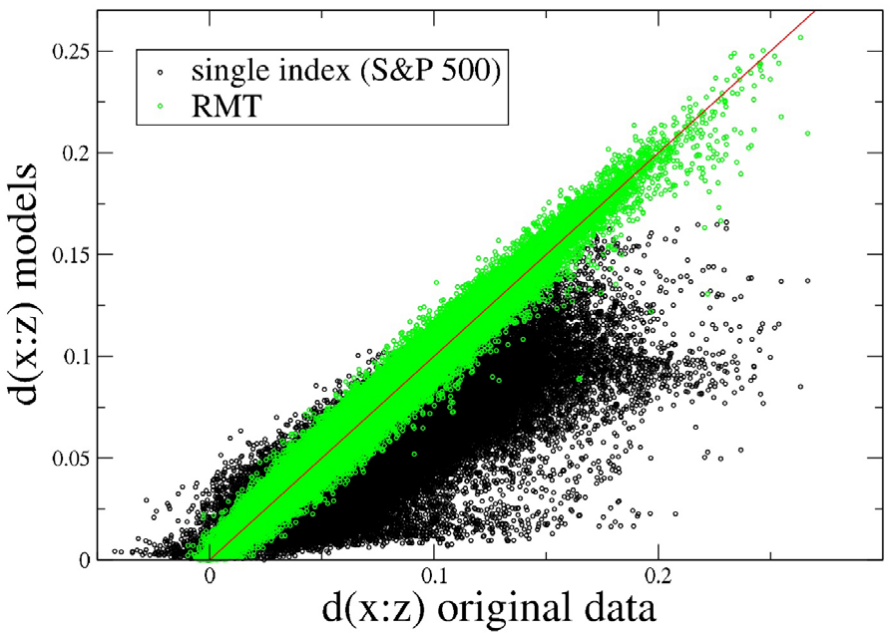
Scatter plot of the quantity 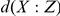 **(see Eq. 4)** as estimated from real data, and as reconstructed by using factor models.

The single index model assumes that linear correlations among the random variables of a system, stock returns in our investigation, are due to the fact that all the variables linearly depend on a single random variable, namely the index. In our comparison of the model with empirical data, we use the daily return of S&P 500 index. In our comparison, we normalize stock returns and S&P 500 return to have zero mean and unit variance. The equation describing the single index model is: 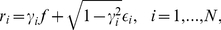
(6)


where 
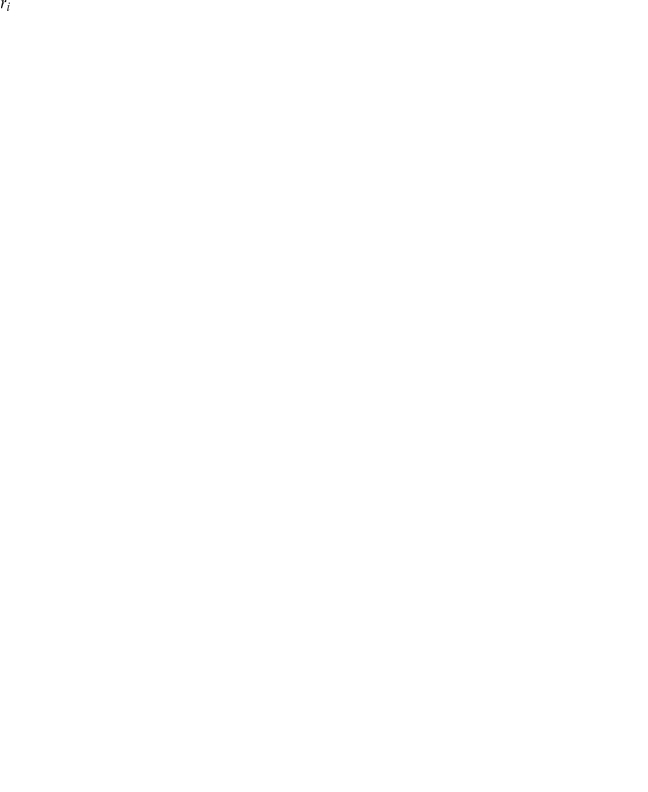
 is the normalized return of stock 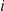, 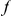 is the return of the index, 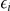 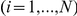 are i.i.d. random variables with zero mean and unit variance, and 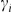 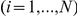 are parameters. The idiosyncratic terms 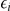 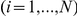 are uncorrelated with 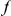. The value of 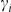 immediately follows from Eq. (6). In fact we have: 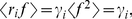
(7)


where we indicate the average of a random variable 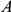 with the symbol 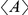. In other words, an estimate of the parameter 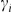 is given by the linear correlation coefficient between the (normalized) stock return 
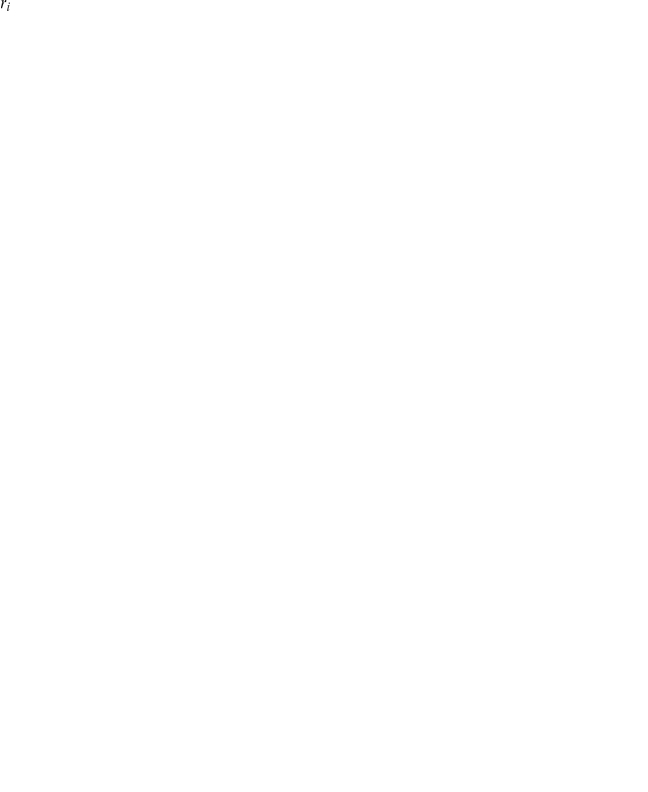
 and the (normalized) index return 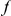. The correlation coefficient 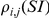 between the variables 
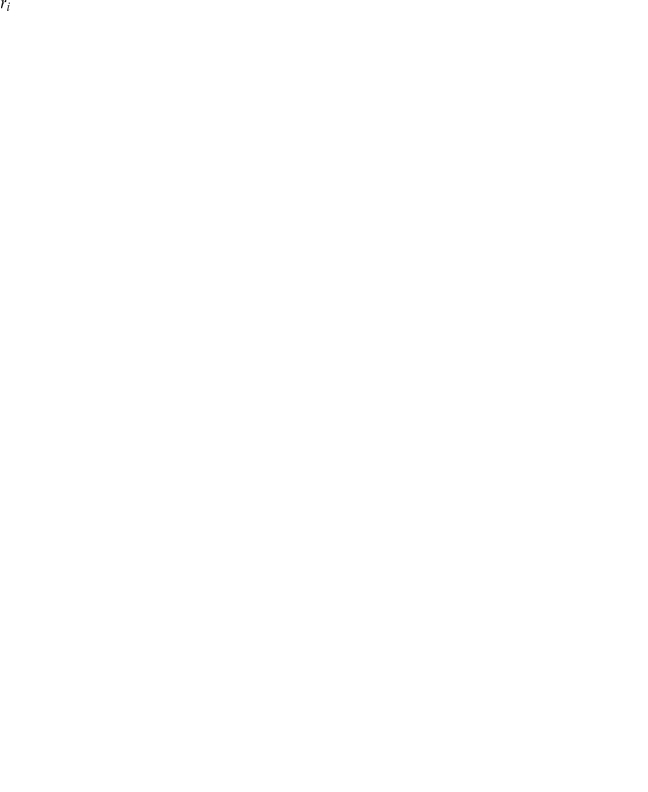
 and 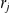 is given by 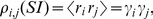
(8)


according to Eq. (6). The linear correlation of Eq. (8) allows one to calculate partial correlations by using Eq. (1), and finally to evaluate the quantities 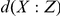 for each pair of elements 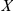 and 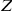 of the system, according to Eq. (4). In Fig. 9, we show a scatter plot of the quantities 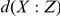 as estimated from real data and from the single index model. It is evident that the single index model poorly reconstructs the empirical values of 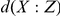, although a trend is present.

#### Model based on RMT

Due to the poor performance of the single index model in reconstructing partial correlations, we consider a more sophisticated model. This model only depends on the properties of the sample correlation matrix 
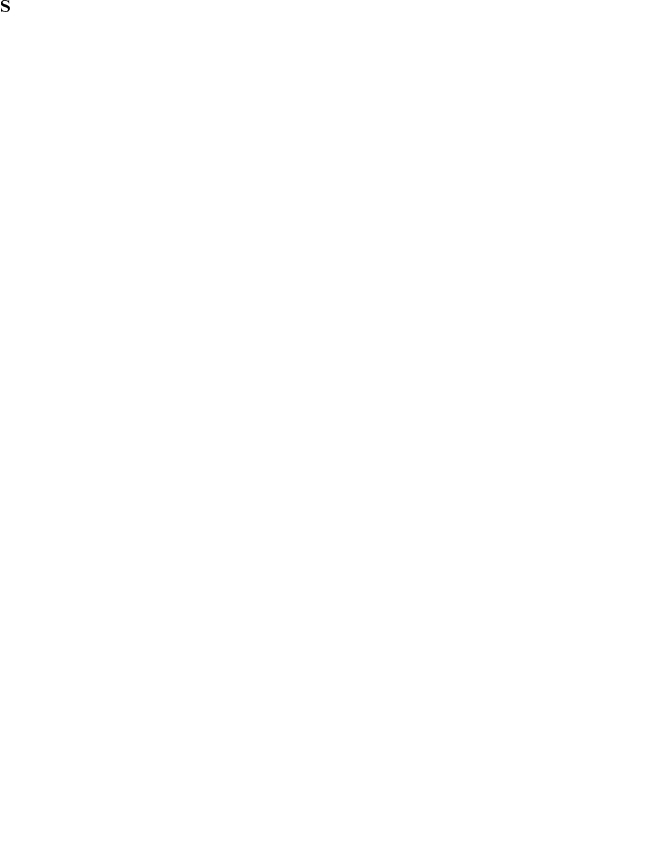
 and the length 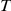 of return time series. Here we again consider normalized stock returns (zero mean and unit variance). The equations of the model are:
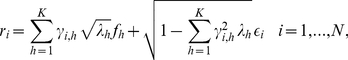
(9)


where 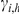 is the 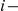
th component of the eigenvector associated with the 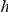-th eigenvalue 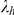 of 
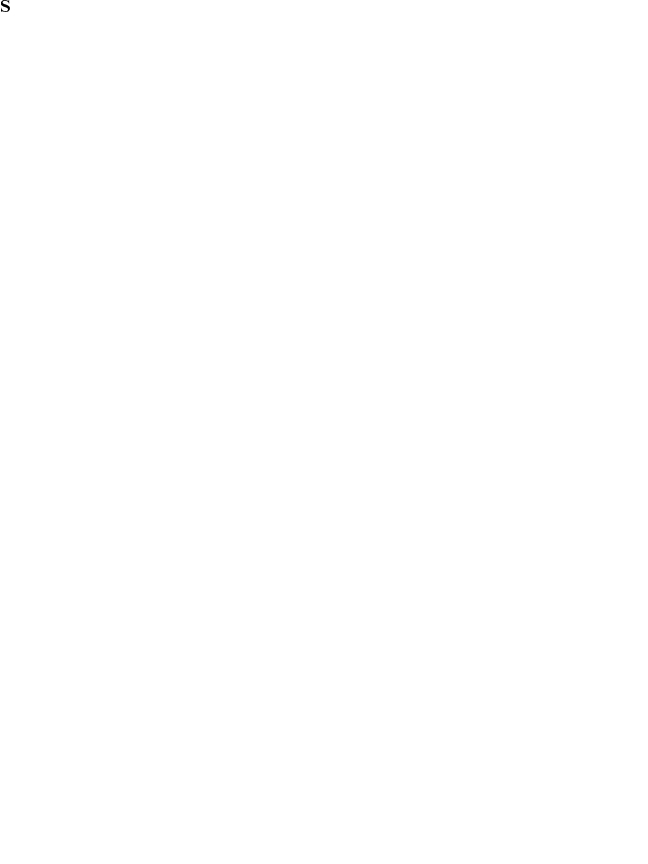
, while 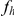 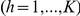 and 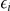 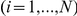 are i.i.d. random variables with zero mean and unit variance [29]. It is assumed that eigenvalues are labeled in decreasing order, i.e. 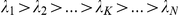. The rationale behind the model is that the economic information carried by the sample correlation matrix 
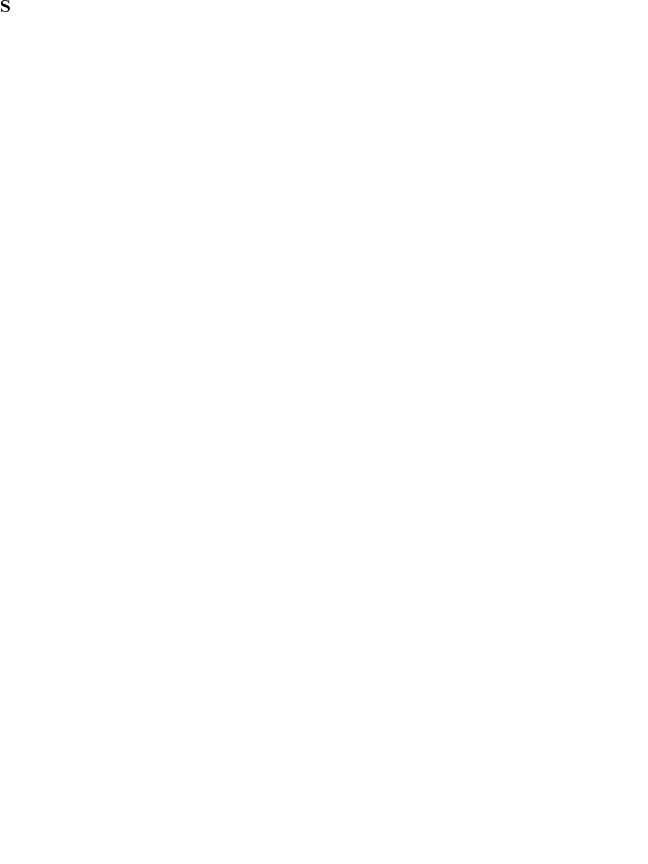
 is mostly present in its largest 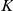 eigenvalues and corresponding eigenvectors. The value of 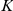 is calculated by comparing the spectrum of 
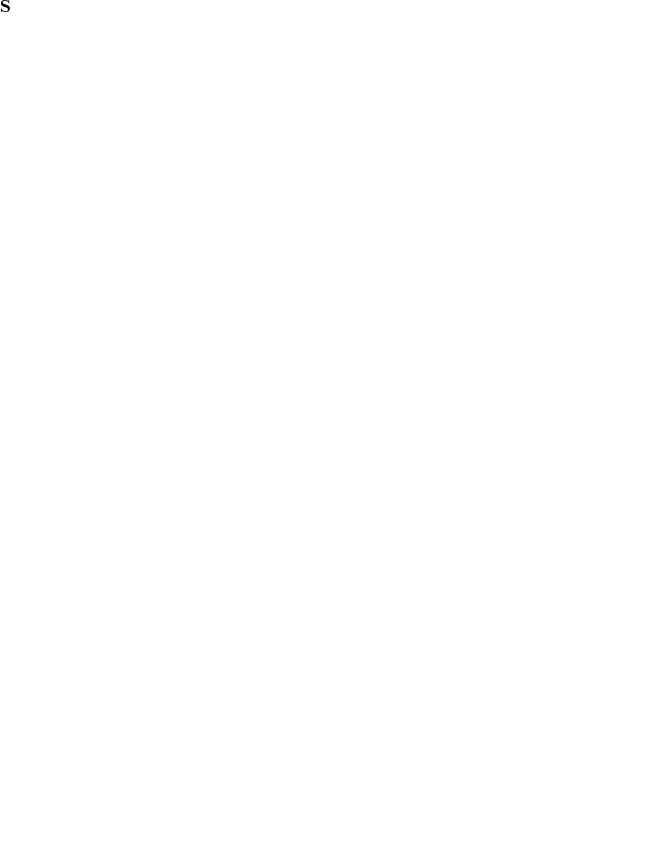
 with the spectrum expected for a random matrix. RMT predicts that the largest eigenvalue of a random matrix [2], [3] cannot be larger than 
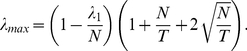
(10)


In our case, 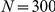, 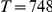, and 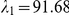. Therefore 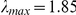. There are 19 eigenvalues of 
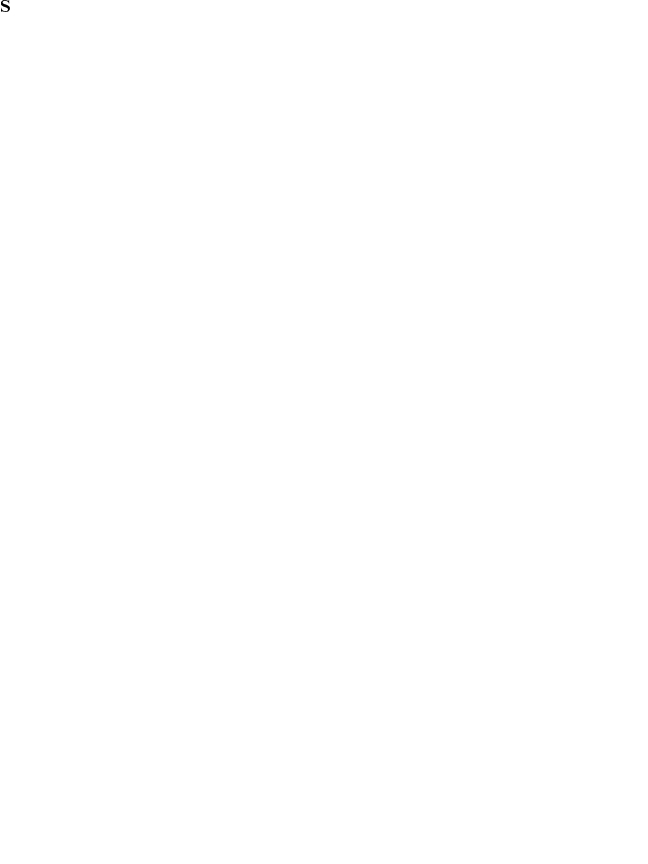
 that are larger than 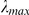, and these eigenvalues explain the 55% of variance of the system. The correlation coefficient 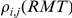 between two variables 
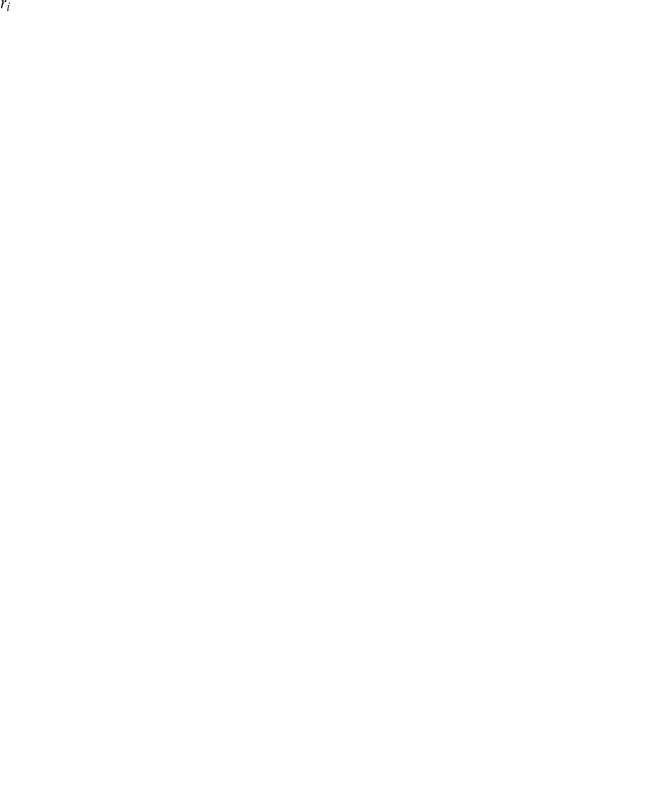
 and 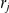 of the system can be calculated from Eq. (9). It results that 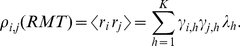
(11)


Also in this case, we can use Eq. (1) to estimate partial correlations for this model, and finally Eq. (4) to evaluate 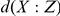 for each pair of elements 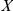 and 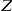 of the system. In Fig. 9, we compare the quantities 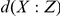 as reconstructed according to this model with 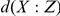 as directly estimated from real data. We observe from this figure that this model provides a rather precise estimation of empirical partial correlations, and the model clearly outperforms the single index model. However the present model involves a large number of factors and parameters. This fact unfortunately prevents a straightforward economic interpretation of the model. The largest eigenvalue 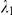 of 
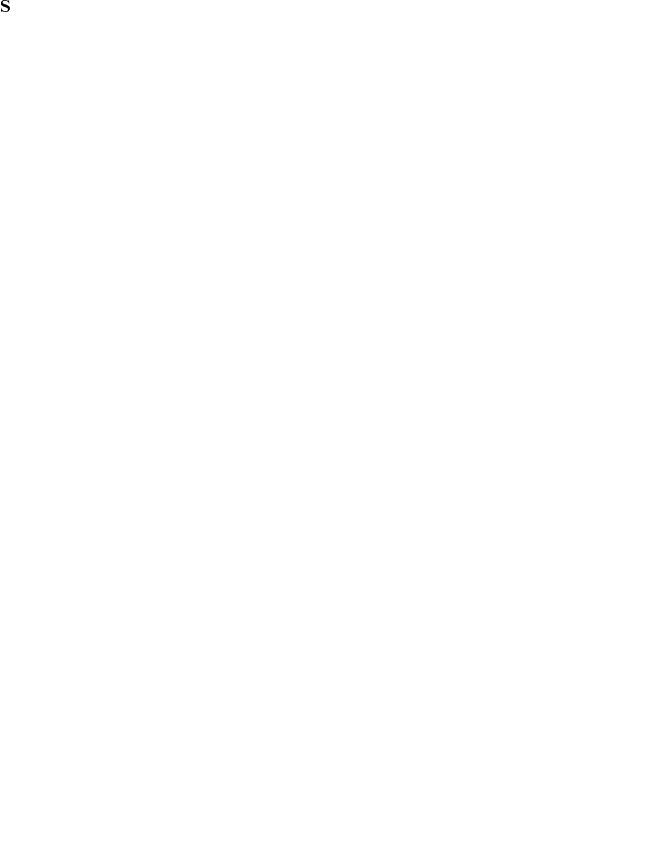
 is associated with the so called market mode, i.e. it represents the tendency of stock returns to follow at some extent the same factor, as it is in the single index model. The remaining 18 eigenvalues with value above the RMT threshold relate to other factors controlling intra-sector correlations and the relation among different economic sectors and sub-sectors. This fact suggests that the clasp of financial sector, as revealed by partial correlation analysis, roots in the properties of the largest 19 eigenvalues and corresponding eigenvectors of the correlation matrix. It is worth noting that no one of these eigenvectors uniquely represents the subspace associated with stocks belonging to the financial sector. This fact suggests that the role of the financial sector in partial correlation networks cannot be simply interpreted as a result of the large average correlation among the stocks belonging to it, but this role is mainly a consequence of inter-sector correlations.

### Dynamical network analysis

**Figure 10:**
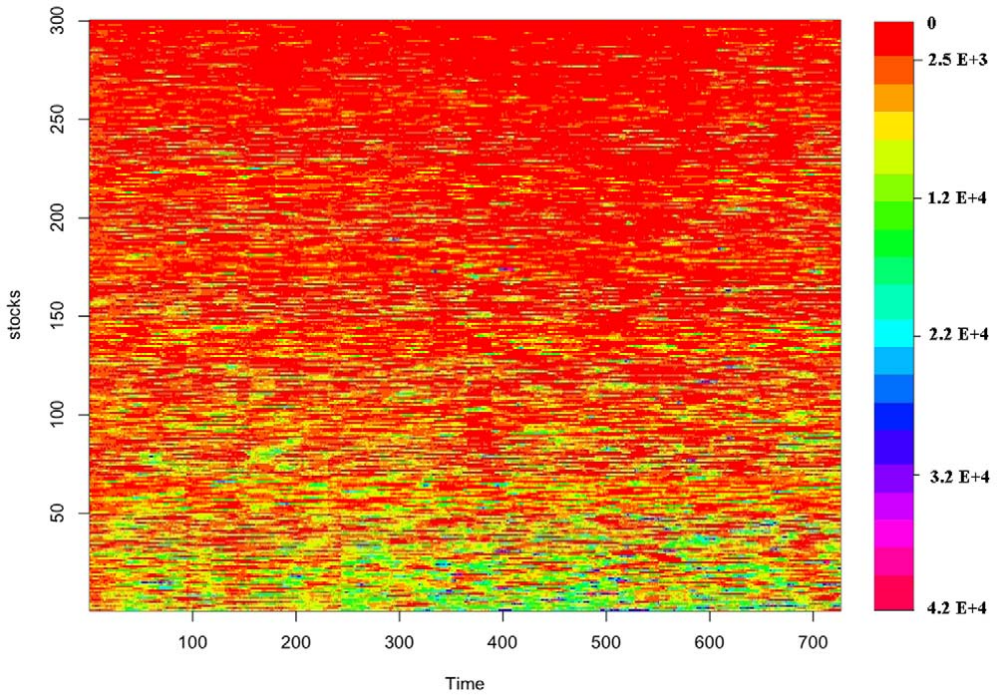
Running window application of the PCTN. Using a 22-day time window, we perform the PCTN in each window, and rank the importance of each stock according to the number of stocks it influenced. The stocks are ordered according to their average influence over time. Most influential stocks are at the bottom of the figure 9 over the 10 most influential stocks are form the financial sector, 5 of them belonging to the sub-sector of investment services.

**Figure 11:**
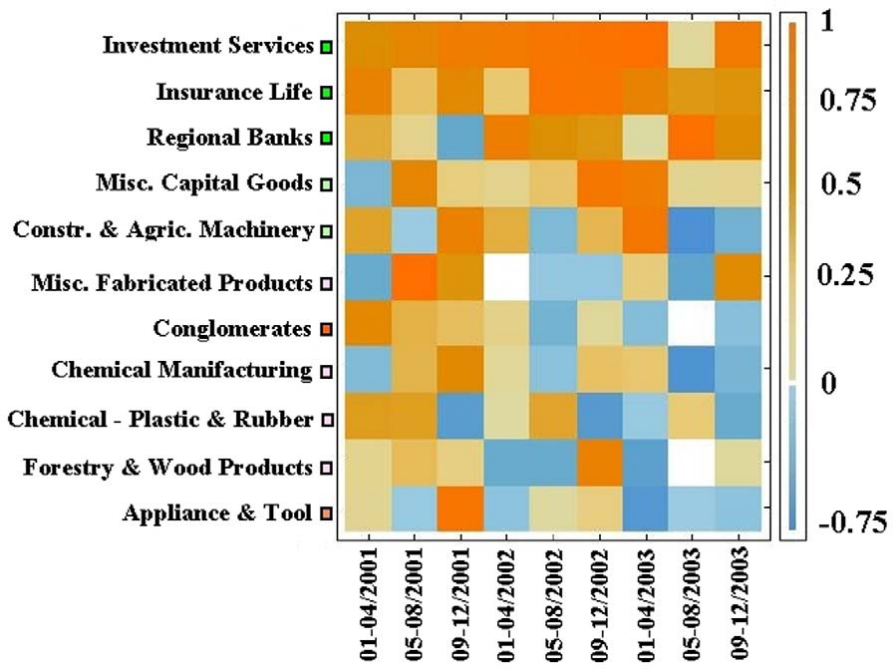
Running window application of the PCPG. Here, each time window corresponds to four months of trading. For each time window we perform the PCPG analysis, and compute the relative influence 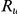 of each economic sub-sector. Here we present the results about 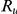 just for the 11 sub-sectors of activity that show a positive relative influence in at least one time window.

We also consider dynamical properties of the PCTN and PCPG by performing the analysis using a moving window approach. This analysis allows one to investigate the stability of the influence of single stocks and groups of stocks across time. We achieve this goal by first making use of a 22-day time window in the PCTN analysis. This small time window allows us to investigate changes of the network at a short time scale, although such a short time window implies noisy estimates of partial correlations. Specifically, at each time window we compute the PCTN, and we use the weighted outdegree 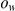 of stocks as a measure of their influence. The results of this investigation are summarized in Fig. 10. While the individual stock importance (the color of each horizontal line in the figure) fluctuates across time, it is possible to observe that highly influential stocks remain so over time. The order of stocks in the figure is given according to their average outdegree over time. Most influential stocks are at the bottom of the figure. The top 10 most influential stocks are BEN (FI, investment services), STI (FI, regional banks), MER (FI, investment services), JP (FI, insurance life), UPC (FI, regional banks), AGE (FI, investment services), LM (FI, investment services), BSC (FI, investment services), CAT (CG, construction & agricultural machinery), and ONE (FI, regional banks). In other words, over the 10 most influential stocks 9 of them are from the financial sector and 5 of them belong to the sub-sector of investment services. In order to check for the influence of specific economic sub-sectors over time we also use the PCPG. In this second investigation, the network is constructed at different periods of time for non-overlapping time windows of four months. The use of a time window of four months has the advantage of increasing the statistical reliability of partial correlation estimates, while considering non-overlapping time windows guaranties independency of influence measures for different time windows. We use different conditions in the PCTN and in the PCPG analysis to evaluate the generality of our results. In the PCPG case, the analysis is performed at the level of economic sub-sectors. For each time window we evaluate the relative influence 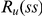 of sub-sectors according to Eq. (5) for each one of the 80 sub-sectors in the system. The results of this analysis are summarized in Fig. 11 for the 11 sub-sectors that show a positive value of 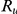 in at least one time window. Once again we find that the economic sub-sector of investment services is the most influential sub-sector in the system. It is to notice that while in the PCTN we were looking at the absolute influence of stocks, i.e. to their weighted outdegree, and despite of their indegree, here we take into account both aspects simultaneously. We can therefore state that the economic sub-sector of investment services is the most influential sub-sector affecting the correlation structure of the entire system, and that this sub-sector is poorly influenced by other economic sub-sectors of activity. The fact that the relative influence of just 11 sub-sectors is positive in at least one time window indicates that most of the 80 economic sub-sectors are more influenced than influential for the correlation structure of the system. This observation makes even more crucial the role of leading sub-sectors like investment services, insurance life, and regional banks.

### Conclusions

We have introduced a network based method to perform partial correlation analysis of multivariate data. We have shown that partial correlation analysis of a financial market suitably complements a correlation based analysis. Indeed our approach is able to detect the prominent role of financial stocks in controlling the correlation structure of the market. A role which is not revealed by standard correlation analysis. Such an influential role of financial stocks is observed at different levels of aggregation of stocks, i.e. it holds true for (i) single stocks, like JP, BEN, and STI, as detailed in Fig. 2, (ii) economic sub-sectors of the financial sector, like investment services, insurance life, and regional banks, as shown in Fig. 3, and (iii) the whole sector of financial stocks, as shown in Fig. 7. The time dependent analysis performed by using moving windows also shows that such an influence of financial stocks is rather stable over time.

## Supporting Information

Table S1 List of 300 stocks
(PDF)

Table S2 List of 80 subsectors
(PDF)
