# Supplementary material for: Dominating Clasp of the Financial Sector Revealed by Partial Correlation Analysis of the Stock Market
Source: PLoS One. 2010 Dec 20;5(12):e15032. doi: 10.1371/journal.pone.0015032 (PMC3004792; doi:10.1371/journal.pone.0015032)
Supplement: Table S1 — List of 300 stocks (PDF) [file pone.0015032.s001.pdf]

index	tick	sector	subsector
1	GE	Conglomerates	Conglomerates
2	PFE	Healthcare	Major Drugs
3	WMT	Services	Retail Department & Discount
4	AIG	Financial	Insurance Prop. & Casualty
5	IBM	Technology	Computer Hardware
6	KO	Consumer_Non_Cyclical	Beverages Non-Alcoholic
7	JNJ	Healthcare	Major Drugs
8	PG	Consumer_Non_Cyclical	Personal & Household Products
9	MRK	Healthcare	Major Drugs
10	BAC	Financial	Money Center Banks
11	WFC	Financial	Money Center Banks
12	SBC	Services	Communication Services
13	FNM	Financial	Consumer Financial Services
14	HD	Services	Retail Home Improvement
15	PEP	Consumer_Non_Cyclical	Beverages Non-Alcoholic
16	LLY	Healthcare	Major Drugs
17	BUD	Consumer_Non_Cyclical	Beverages Alcoholic
18	ABT	Healthcare	Major Drugs
19	BMY	Healthcare	Major Drugs
20	AXP	Financial	Consumer Financial Services
21	MER	Financial	Investment Services
22	MDT	Healthcare	Medical Equipment & Supplies
23	UTX	Conglomerates	Conglomerates
24	BLS	Services	Communication Services
25	ONE	Financial	Regional Banks
26	TYC	Conglomerates	Conglomerates
27	TXN	Technology	Semiconductors
28	G	Consumer_Non_Cyclical	Personal & Household Products
29	DD	Basic_Materials	Chemical - Plastic & Rubber
30	DIS	Services	Broadcasting & Cable TV
31	LOW	Services	Retail Home Improvement
32	BA	Capital_Good	Aerospace & Defense
33	FRE	Financial	Consumer Financial Services
34	MCD	Services	Restaurants
35	DOW	Basic_Materials	Chemical - Plastic & Rubber
36	GM	Consumer_Cyclical	Auto & Truck Manufacturers
37	ALL	Financial	Insurance Prop. & Casualty
38	WAG	Services	Retail Drugs
39	FDC	Technology	Computer Services
40	CL	Consumer_Non_Cyclical	Personal & Household Products
41	SLB	Energy	Oil Well Services & Equipment
42	SGP	Healthcare	Major Drugs
43	BK	Financial	Money Center Banks
44	CAT	Capital_Good	Constr. & Agric. Machinery
45	KMB	Basic_Materials	Paper & Paper Products
46	MOT	Technology	Communication Equipment
47	KRB	Financial	Regional Banks
48	EMR	Conglomerates	Conglomerates
49	BSX	Healthcare	Medical Equipment & Supplies
50	EMC	Technology	Computer Storage Devices
51	GCI	Services	Printing & Publishing
52	CCU	Services	Broadcasting & Cable TV

53	CAH	Healthcare	Biotechnology & Drugs
54	SYY	Services	Retail Grocery
55	TRB	Services	Printing & Publishing
56	MMC	Financial	Insurance Miscellaneous
57	RD	Energy	Oil & Gas - Integrated
58	ITW	Capital_Good	Misc. Capital Goods
59	IGT	Services	Casinos & Gaming
60	AVP	Consumer_Non_Cyclical	Personal & Household Products
61	AFL	Financial	Insurance Accidental & Health
62	GIS	Consumer_Non_Cyclical	Food Processing
63	GPS	Services	Retail Apparel
64	NCC	Financial	Regional Banks
65	SO	Utilities	Electric Utilities
66	GD	Capital_Good	Aerospace & Defense
67	STI	Financial	Regional Banks
68	IP	Basic_Materials	Paper & Paper Products
69	LEH	Financial	Investment Services
70	BAX	Healthcare	Medical Equipment & Supplies
71	S	Services	Services
72	PNC	Financial	Regional Banks
73	PGR	Financial	Insurance Prop. & Casualty
74	UNP	Transportation	Railroad
75	MEL	Financial	Investment Services
76	GDT	Healthcare	Medical Equipment & Supplies
77	PPG	Basic_Materials	Chemical Manifacturing
78	DUK	Utilities	Electric Utilities
79	NEM	Basic_Materials	Gold & Silver
80	NOC	Capital_Good	Aerospace & Defense
81	DE	Capital_Good	Constr. & Agric. Machinery
82	OMC	Services	Advertising
83	CA	Technology	Software & Programming
84	SLE	Consumer_Non_Cyclical	Food Processing
85	ADI	Technology	Semiconductors
86	BBY	Services	Retail Technology
87	KR	Services	Retail Grocery
88	SCH	Financial	Investment Services
89	OXY	Energy	Oil & Gas Operations
90	BNI	Transportation	Railroad
91	K	Consumer_Non_Cyclical	Food Processing
92	CI	Financial	Insurance Accidental & Health
93	GDW	Financial	S&Ls/Savings Banks
94	HDI	Consumer_Cyclical	Recreational Products
95	DHR	Technology	Scientific & Technical Instr.
96	KSS	Services	Retail Department & Discount
97	WLP	Financial	Insurance Accidental & Health
98	CAG	Consumer_Non_Cyclical	Food Processing
99	FON	Services	Communication Services
100	BSC	Financial	Investment Services
101	AT	Services	Communication Services
102	CPB	Consumer_Non_Cyclical	Food Processing
103	GLW	Technology	Communication Equipment
104	WPO	Services	Printing & Publishing
105	KEY	Financial	Regional Banks

106	MHP	Services	Printing & Publishing
107	ETR	Utilities	Electric Utilities
108	WY	Basic_Materials	Forestry & Wood Products
109	COF	Financial	Regional Banks
110	BDX	Healthcare	Medical Equipment & Supplies
111	PX	Basic_Materials	Chemical Manifacturing
112	APA	Energy	Oil & Gas Operations
113	CB	Financial	Insurance Prop. & Casualty
114	BR	Energy	Oil & Gas Operations
115	APD	Basic_Materials	Chemical Manifacturing
116	PBI	Technology	Office Equipment
117	APC	Energy	Oil & Gas Operations
118	HNZ	Consumer_Non_Cyclical	Food Processing
119	MAS	Consumer_Cyclical	Furniture & Fixtures
120	BEN	Financial	Investment Services
121	LUV	Transportation	Airline
122	SWY	Services	Retail Grocery
123	FD	Services	Retail Department & Discount
124	AEP	Utilities	Electric Utilities
125	CLX	Consumer_Non_Cyclical	Personal & Household Products
126	HRB	Services	Personal Services
127	FPL	Utilities	Electric Utilities
128	HAL	Energy	Oil Well Services & Equipment
129	MAR	Services	Hotels & Motels
130	HSY	Consumer_Non_Cyclical	Food Processing
131	NKE	Consumer_Cyclical	Footwear
132	TXT	Conglomerates	Conglomerates
133	TJX	Services	Retail Apparel
134	XRX	Technology	Office Equipment
135	BHI	Energy	Oil Well Services & Equipment
136	WWY	Consumer_Non_Cyclical	Food Processing
137	TMX	Services	Communication Services
138	JCI	Consumer_Cyclical	Auto & Truck Parts
139	ROH	Basic_Materials	Chemical - Plastic & Rubber
140	AGN	Healthcare	Biotechnology & Drugs
141	ASO	Financial	Regional Banks
142	ADM	Consumer_Non_Cyclical	Food Processing
143	CCE	Consumer_Non_Cyclical	Beverages Non-Alcoholic
144	EK	Consumer_Cyclical	Photography
145	CMA	Financial	Regional Banks
146	PEG	Utilities	Electric Utilities
147	UCL	Energy	Oil & Gas Operations
148	NSC	Transportation	Railroad
149	MCK	Consumer_Non_Cyclical	Personal & Household Products
150	DOV	Conglomerates	Conglomerates
151	LTD	Services	Retail Apparel
152	LTR	Financial	Insurance Prop. & Casualty
153	ETN	Capital_Good	Misc. Capital Goods
154	MBI	Financial	Insurance Prop. & Casualty
155	SPC	Technology	Technology
156	SNV	Financial	Regional Banks
157	SPG	Services	Real Estate Operations
158	AZO	Services	Retail Specialty

159	MAT	Consumer_Cyclical	Recreational Products
160	ECL	Consumer_Non_Cyclical	Personal & Household Products
161	ABS	Services	Retail Grocery
162	CSC	Technology	Computer Services
163	YPF	Energy	Oil & Gas Operations
164	MU	Technology	Semiconductors
165	EQR	Services	Real Estate Operations
166	PD	Basic_Materials	Metal Mining
167	AOC	Financial	Insurance Miscellaneous
168	CSX	Transportation	Railroad
169	GP	Basic_Materials	Paper & Paper Products
170	ROK	Technology	Electronics Instruments & Controls
171	MYL	Healthcare	Biotechnology & Drugs
172	ABK	Financial	Insurance Prop. & Casualty
173	UST	Consumer_Non_Cyclical	Tobacco
174	SHW	Services	Retail Home Improvement
175	LNC	Financial	Insurance Life
176	JCP	Services	Retail Department & Discount
177	JP	Financial	Insurance Life
178	NSM	Technology	Semiconductors
179	NFB	Financial	Regional Banks
180	PH	Basic_Materials	Misc. Fabricated Products
181	AVY	Basic_Materials	Container & Packaging
182	MTG	Financial	Insurance Prop. & Casualty
183	ABX	Basic_Materials	Gold & Silver
184	UN	Consumer_Non_Cyclical	Food Processing
185	CIN	Utilities	Electric Utilities
186	CTX	Capital_Good	Construction Services
187	RCL	Services	Recreational Activities
188	TOT	Energy	Oil & Gas - Integrated
189	FDO	Services	Retail Specialty
190	ODP	Services	Retail Specialty
191	HLT	Services	Hotels & Motels
192	VMC	Capital_Good	Construction - Raw Materials
193	TIF	Services	Retail Specialty
194	NWL	Consumer_Non_Cyclical	Personal & Household Products
195	WHR	Consumer_Cyclical	Appliance & Tool
196	HMA	Healthcare	Healthcare Facilities
197	MGG	Services	Casinos & Gaming
198	LIZ	Consumer_Cyclical	Apparel/Accessories
199	SSP	Services	Printing & Publishing
200	BJS	Energy	Oil Well Services & Equipment
201	KRI	Services	Printing & Publishing
202	VNO	Services	Real Estate Operations
203	MUR	Energy	Oil & Gas Operations
204	TSS	Technology	Computer Services
205	IPG	Services	Advertising
206	VFC	Consumer_Cyclical	Apparel/Accessories
207	UPC	Financial	Regional Banks
208	GGP	Services	Real Estate Operations
209	HAR	Consumer_Cyclical	Audio & Video Equipment
210	GPC	Consumer_Cyclical	Auto & Truck Parts
211	PHM	Capital_Good	Construction Services

212	PCL	Basic_Materials	Forestry & Wood Products
213	VLO	Energy	Oil & Gas Operations
214	TER	Technology	Semiconductors
215	TMK	Financial	Insurance Accidental & Health
216	LUK	Conglomerates	Conglomerates
217	CNA	Financial	Insurance Prop. & Casualty
218	FNF	Financial	Insurance Prop. & Casualty
219	WMB	Utilities	Natural Gas Utilities
220	AMD	Technology	Semiconductors
221	JNY	Consumer_Cyclical	Apparel/Accessories
222	GWW	Capital_Good	Misc. Capital Goods
223	LM	Financial	Investment Services
224	NUE	Basic_Materials	Iron & Steel
225	HB	Technology	Scientific & Technical Instr.
226	UIS	Technology	Computer Services
227	KIM	Services	Real Estate Operations
228	SLR	Technology	Electronics Instruments & Controls
229	AHC	Energy	Oil & Gas - Integrated
230	CDN	Technology	Software & Programming
231	TCB	Financial	Regional Banks
232	BCR	Healthcare	Medical Equipment & Supplies
233	KMG	Energy	Oil & Gas Operations
234	CTL	Services	Communication Services
235	UNM	Financial	Insurance Accidental & Health
236	VAR	Healthcare	Medical Equipment & Supplies
237	ORI	Financial	Insurance Prop. & Casualty
238	RHI	Services	Services
239	BLL	Basic_Materials	Container & Packaging
240	SEE	Basic_Materials	Container & Packaging
241	GTK	Technology	Computer Services
242	WEN	Services	Restaurants
243	SFA	Technology	Communication Equipment
244	EFX	Services	Business Services
245	EC	Basic_Materials	Chemical Manifacturing
246	LEA	Consumer_Cyclical	Auto & Truck Parts
247	MME	Healthcare	Healthcare
248	TMO	Technology	Scientific & Technical Instr.
249	SBL	Technology	Computer Peripherals
250	SVU	Services	Retail Grocery
251	TRH	Financial	Insurance Accidental & Health
252	GR	Capital_Good	Aerospace & Defense
253	LEG	Consumer_Cyclical	Furniture & Fixtures
254	SII	Energy	Oil Well Services & Equipment
255	DNY	Services	Services
256	DRE	Services	Real Estate Operations
257	OCR	Services	Retail Drugs
258	MAN	Services	Services
259	SNE	Consumer_Cyclical	Audio & Video Equipment
260	DJ	Services	Printing & Publishing
261	IFF	Basic_Materials	Chemical Manifacturing
262	N	Basic_Materials	Metal Mining
263	DBD	Technology	Office Equipment
264	HUM	Financial	Insurance Accidental & Health

265	HIB	Financial	Regional Banks
266	RBK	Consumer_Cyclical	Footwear
267	HMT	Services	Real Estate Operations
268	WEC	Utilities	Electric Utilities
269	EAT	Services	Restaurants
270	TIN	Conglomerates	Conglomerates
271	BDK	Consumer_Cyclical	Appliance & Tool
272	TOY	Services	Retail Specialty
273	NWS	Services	Printing & Publishing
274	BFB	Consumer_Non_Cyclical	Beverages Alcoholic
275	SVM	Services	Services
276	PNW	Utilities	Electric Utilities
277	NAV	Consumer_Cyclical	Auto & Truck Manufacturers
278	TV	Services	Broadcasting & Cable TV
279	HRL	Consumer_Non_Cyclical	Food Processing
280	TEF	Services	Communication Services
281	SWK	Consumer_Cyclical	Appliance & Tool
282	AGE	Financial	Investment Services
283	PLL	Technology	Scientific & Technical Instr.
284	APH	Technology	Electronics Instruments & Controls
285	DPL	Utilities	Electric Utilities
286	PVN	Financial	Regional Banks
287	MDP	Services	Printing & Publishing
288	EMN	Basic_Materials	Chemical - Plastic & Rubber
289	LSI	Technology	Semiconductors
290	BCE	Services	Communication Services
291	AIV	Services	Real Estate Operations
292	VSH	Technology	Electronics Instruments & Controls
293	BEC	Technology	Scientific & Technical Instr.
294	BC	Consumer_Cyclical	Recreational Products
295	MYG	Consumer_Cyclical	Appliance & Tool
296	HCP	Services	Real Estate Operations
297	EQT	Utilities	Natural Gas Utilities
298	IRF	Technology	Semiconductors
299	CYN	Financial	Regional Banks
300	AL	Basic_Materials	Metal Mining

company_name
General Electric
PFIZER INC
WAL-MART STORES INC
AMERICAN INTL GROUP INC
INTL BUSINESS MACHINES CORP
COCA-COLA CO
JOHNSON AND JOHNSON
PROCTER   GAMBLE CO
MERCK   CO INC
BANK OF AMERICA CORP
WELLS FARGO   CO NEW
SBC COMMUNICATIONS INC
FANNIE MAE
HOME DEPOT INC
PEPSICO INC
LILLY ELI   CO
ANHEUSER BUSCH COS INC
ABBOTT LABORATORIES
BRISTOL MYERS SQUIBB COMPANY
AMERICAN EXPRESS COMPANY
MERRILL LYNCH   CO INC
MEDTRONIC INC
UNITED TECHNOLOGIES CORP
BELLSOUTH CORPORATION
BANK ONE CORP
TYCO INTERNATIONAL LTD NEW
TEXAS INSTRUMENTS
GILLETTE CO
DU PONT DE NEMOURS E I CO
WALT DISNEY CO-DISNEY COMMON
LOWES COMPANIES INC
BOEING CO
FREDDIE MAC D/B/A VOTING
MCDONALDS CORP
DOW CHEMICAL CO
GENERAL MOTORS CORP
ALLSTATE CORP THE
WALGREEN COMPANY
FIRST DATA CORP
COLGATE-PALMOLIVE CO
SCHLUMBERGER LTD
SCHERING PLOUGH CORP
BANK OF NEW YORK INC
CATERPILLAR INC
KIMBERLY CLARK CORP
MOTOROLA INC
M B N A CORP
EMERSON ELECTRIC CO
BOSTON SCIENTIFIC CORP
EMC CORPORATION
GANNETT CO INC
CLEAR CHANNEL COMMUNICTNS INC

CARDINAL HEALTH INC
SYSCO CORP
TRIBUNE COMPANY
MARSH MCLENNAN COS INC
ROYAL DUTCH PET NEW 1.25GLDRS
ILLINOIS TOOL WORKS
INTL GAME TECHNOLOGY
AVON PRODUCTS INC
AFLAC INC
GENERAL MILLS INC
GAP INC THE
NATIONAL CITY CORP
SOUTHERN CO
GENERAL DYNAMICS CORP
SUNTRUST BANKS INC
INTERNATIONAL PAPER CO
LEHMAN BROTHERS HOLDINGS
BAXTER INTERNATIONAL INC
SEARS ROEBUCK CO
PNC FINL SVCS GRP INC THE
PROGRESSIVE CORP
UNION PACIFIC CORPORATION
MELLON FINANCIAL CORP
GUIDANT CORP
PPG INDUSTRIES INC
DUKE ENERGY CORPORATION
NEWMONT MINING CORP HOLDING C
NORTHROP GRUMMAN CP HLDG CO
DEERE CO
OMNICOM GROUP INC
COMPUTER ASSOCIATES INTL INC
SARA LEE CORP
ANALOG DEVICES INC
BEST BUY CO INC
KROGER CO
SCHWAB CHARLES CORP
OCCIDENTAL PETROLEUM CORP
BURLINGTON NRTHRN SANTA FE COM
KELLOGG CO
CIGNA CORP
GOLDEN WEST FINANCIAL CORP
HARLEY DAVIDSON INC
DANAHER CORP
KOHLS CORP
WELLPOINT HLTH NETWKS HLDG CO
CONAGRA FOODS INC.
SPRINT CORP FON GROUP
BEAR STEARNS COMPANIES INC
ALLTEL CORP
CAMPBELL SOUP CO CAPITAL
CORNING INCORPORATED
WASHINGTON POST CO CLB
KEYCORP NEW

MCGRAW-HILL COS INC THE
ENTERGY CORP NEW
WEYERHAEUSER COMPANY
CAPITAL ONE FINANCIAL CORP
BECTON DICKINSON   CO
PRAXAIR INC
APACHE CORP
CHUBB CORPORATION
BURLINGTON RESOURCES INC
AIR PROD CHEM INC
PITNEY-BOWES INC
ANADARKO PETROLEUM CORP
HEINZ H.J. CO
MASCO CORP
FRANKLIN RESOURCES
SOUTHWEST AIRLINES CO
SAFEWAY INC
FEDERATED DEPRTMNT STORES NEW
AMERICAN ELECTRIC POWER INC
CLOROX CO
BLOCK H   R INC
FPL GROUP INC
HALLIBURTON CO HOLDING CO
MARRIOTT INT L INC NEW CL A
HERSHEY FOODS CORP
NIKE INC CL-B
TEXTRON INCORPORATED OF DEL
TJX COMPANIES INC
XEROX CORPORATION
BAKER HUGHES INC
WRIGLEY WILLIAM JR
TELEFONOS DE MEX ADR REP 20-L
JOHNSON CONTROLS INC
ROHM AND HASS COMPANY
ALLERGAN INC
AMSOUTH BANCORPORATION
ARCHER DANIELS MIDLAND CO
COCA-COLA ENTERPRISES INC.
EASTMAN KODAK CO
COMERICA INC
PUB SVC ENTERPRISE GROUP INC
UNOCAL CORP DEL
NORFOLK SOUTHERN CORP
MCKESSON CORPORATION
DOVER CORP
LIMITED BRANDS INC.
LOEWS CORP
EATON CORP
MBIA INC
ST PAUL CO S INC THE
SYNOVUS FINANCIAL CORP
SIMON PROPERTIES GROUP INC
AUTOZONE INC

MATTEL INC
ECOLAB INC
ALBERTSON S INC
COMPUTER SCIENCES CORP
YPF SOCIEDAD ANONIMA ADS
MICRON TECHNOLOGY INC
EQUITY RESIDENTIAL
PHELPS DODGE CORP
AON CORPORATION
CSX CORP
GEORGIA PACIFIC CORP
ROCKWELL AUTOMATION INC.
MYLAN LABORATORIES INC
AMBAC FINANCIAL GROUP INC.
UST INC
SHERWIN-WILLIAMS CO
LINCOLN NATIONAL CORP
PENNEY J.C. CO INC HOLDING CO
JEFFERSON-PILOT CORP
NATIONAL SEMICONDUCTOR CORP
NORTH FORK BANCORPORATION INC
PARKER-HANNIFIN CORP
AVERY DENNISON CORP
MGIC INVESTMENT CORP
BARRICK GOLD CORP
UNILEVER NV NEW NY SHARES
CINERGY CORP
CENTEX CORP
ROYAL CARIBBEAN CRUISES LTD
TOTAL S.A. ADS REPTG 1/2 OF A
FAMILY DOLLAR STORES
OFFICE DEPOT INC
HILTON HOTELS CORP
VULCAN MATERIALS COMPANY
TIFFANY CO
NEWELL RUBBERMAID INC
WHIRLPOOL CORPORATION
HEALTH MGMT ASSOC INC CL-A
MGM MIRAGE
LIZ CLAIBORNE INC
SCRIPPS CO E.W. CL-A NEW
BJ SERVICES CO
KNIGHT-RIDDER INC
VORNADO REALTY TRUST
MURPHY OIL CORPORATION
TOTAL SYSTEM SERVICES INC
INTERPUBLIC GROUP COS INC
V. F. CORPORATION
UNION PLANTERS CORP
GENERAL GROWTH PROPERTIES INC
HARMAN INT L IND INC
GENUINE PARTS CO
PULTE HOMES INC

PLUM CREEK TIMBER CO REIT
VALERO ENERGY CORP NEW
TERADYNE INC
TORCHMARK CORP
LEUCADIA NATIONAL CORP
CNA FINANCIAL CORP
FIDELITY NATL FINANCIAL INC
WILLIAMS COMPANIES
ADVANCED MICRO DEVICES
JONES APPAREL GROUP INC
GRAINGER W.W.INC
LEGG MASON INC
NUCOR CORP
HILLEBRAND INDUSTRIES INC
UNISYS CORP
KIMCO REALTY CORPORATION
SOLECTRON CORP
AMERADA HESS CORP
CADENCE DESIGN SYS INC
TCF FINANCIAL CORP
BARD C R INC
KERR-MCGEE CORP HOLDING COMPA
CENTURYTEL INC
UNUMPROVIDENT CRP
VARIAN MEDICAL SYSTEMS INC
OLD REPUBLIC INTL CORP
ROBERT HALF INTERNATIONAL INC
BALL CORP
SEALED AIR CORP NEW
GTECH HOLDINGS CORP
WENDYS INTERNATIONAL
SCIENTIFIC ATLANTA INC
EQUIFAX INCORPORATED
ENGELHARD CORP
LEAR CORP
MID ATLANTIC MEDICAL SVCS INC
THERMO ELECTRON CORP
SYMBOL TECHNOLOGIES INC
SUPERVALU INC
TRANSATLANTIC HOLDINGS INC
GOODRICH CORP
LEGGETT PLATT INC
SMITH INTERNATIONAL INC
DONNELLEY R R   SONS CO
DUKE REALTY CORP
OMNICARE INC
MANPOWER INC
SONY CORP ADR
DOW JONES COMPANY INC
INTL FLAVORS   FRAGRANCES
INCO LIMITED
DIEBOLD INC
HUMANA INC

HIBERNIA CORP CL A VOTING
REEBOK INTERNATIONAL LTD
HOST MARRIOTT CORP REIT
WISCONSIN ENERGY CORP
BRINKER INTL INC
TEMPLE INLAND INC
BLACK DECKER CORP
TOYS R US INC HLDG CO
NEWS CORP LTD ADS
BROWN-FORMAN CORP CL-B
SERVICEMASTER CO THE
PINNACLE WEST CAP CORP
NAVISTAR INTL CORP HLDG CO
GRUPO TELEVISA SA GDS RP20 ORD
HORMEL FOODS CORPORATION
TELEFONICA S.A.ADS RP 3 SHS
STANLEY WORKS
A.G.EDWARDS INC
PALL CORP
AMPHENOL CORP NEW CL-A
DPL INC HOLDING CO
PROVIDIAN FINANCIAL BANCORP
MEREDITH CORP
EASTMAN CHEMICAL CO
LSI LOGIC CORPORATION
BCE INC
APARTMENT INVST MGMT CO
VISHAY INTERTECHNOLOGY INC
BECKMAN COULTER INC.
BRUNSWICK CORP
MAYTAG CORPORATION
HEALTH CARE PROPERTY INVS INC
EQUITABLE RESOURCES INC
INTL RECTIFIER CORP
CITY NATIONAL CORPORATION
ALCAN INC
