# Supplementary material for: Dominating Clasp of the Financial Sector Revealed by Partial Correlation Analysis of the Stock Market
Source: PLoS One. 2010 Dec 20;5(12):e15032. doi: 10.1371/journal.pone.0015032 (PMC3004792; doi:10.1371/journal.pone.0015032)
Supplement: Table S2 — List of 80 subsectors (PDF) [file pone.0015032.s002.pdf]

index	subsector	sector	#_stocks
1	Advertising	Services	2
2	Aerospace & Defense	Capital_Good	4
3	Airline	Transportation	1
4	Apparel/Accessories	Consumer_Cyclical	3
5	Appliance & Tool	Consumer_Cyclical	4
6	Audio & Video Equipment	Consumer_Cyclical	2
7	Auto & Truck Manufacturers	Consumer_Cyclical	2
8	Auto & Truck Parts	Consumer_Cyclical	3
9	Beverages Alcoholic	Consumer_Non_Cyclical	2
10	Beverages Non-Alcoholic	Consumer_Non_Cyclical	3
11	Biotechnology & Drugs	Healthcare	3
12	Broadcasting & Cable TV	Services	3
13	Business Services	Services	1
14	Casinos & Gaming	Services	2
15	Chemical Manifacturing	Basic_Materials	5
16	Chemical - Plastic & Rubber	Basic_Materials	4
17	Communication Equipment	Technology	3
18	Communication Services	Services	8
19	Computer Hardware	Technology	1
20	Computer Peripherals	Technology	1
21	Computer Services	Technology	5
22	Computer Storage Devices	Technology	1
23	Conglomerates	Conglomerates	8
24	Constr. & Agric. Machinery	Capital_Good	2
25	Construction - Raw Materials	Capital_Good	1
26	Construction Services	Capital_Good	2
27	Consumer Financial Services	Financial	3
28	Container & Packaging	Basic_Materials	3
29	Electric Utilities	Utilities	10
30	Electronics Instruments & Controls	Technology	4
31	Food Processing	Consumer_Non_Cyclical	11
32	Footwear	Consumer_Cyclical	2
33	Forestry & Wood Products	Basic_Materials	2
34	Furniture & Fixtures	Consumer_Cyclical	2
35	Gold & Silver	Basic_Materials	2
36	Healthcare	Healthcare	1
37	Healthcare Facilities	Healthcare	1
38	Hotels & Motels	Services	2
39	Insurance Accidental & Health	Financial	7
40	Insurance Life	Financial	2
41	Insurance Miscellaneous	Financial	2
42	Insurance Prop. & Casualty	Financial	11
43	Investment Services	Financial	8
44	Iron & Steel	Basic_Materials	1
45	Major Drugs	Healthcare	7
46	Medical Equipment & Supplies	Healthcare	7
47	Metal Mining	Basic_Materials	3
48	Misc. Capital Goods	Capital_Good	3
49	Misc. Fabricated Products	Basic_Materials	1
50	Money Center Banks	Financial	3
51	Natural Gas Utilities	Utilities	2
52	Office Equipment	Technology	3

53	Oil & Gas - Integrated	Energy	3
54	Oil & Gas Operations	Energy	9
55	Oil Well Services & Equipment	Energy	5
56	Paper & Paper Products	Basic_Materials	3
57	Personal & Household Products	Consumer_Non_Cyclical	8
58	Personal Services	Services	1
59	Photography	Consumer_Cyclical	1
60	Printing & Publishing	Services	9
61	Railroad	Transportation	4
62	Real Estate Operations	Services	9
63	Recreational Activities	Services	1
64	Recreational Products	Consumer_Cyclical	3
65	Regional Banks	Financial	16
66	Restaurants	Services	3
67	Retail Apparel	Services	3
68	Retail Department & Discount	Services	4
69	Retail Drugs	Services	2
70	Retail Grocery	Services	5
71	Retail Home Improvement	Services	3
72	Retail Specialty	Services	5
73	Retail Technology	Services	1
74	Scientific & Technical Instr.	Technology	5
75	Semiconductors	Technology	8
76	Services	Services	5
77	S&Ls/Savings Banks	Financial	1
78	Software & Programming	Technology	2
79	Technology	Technology	1
80	Tobacco	Consumer_Non_Cyclical	1
